# Vertically Transported Graphene Oxide for High‐Performance Osmotic Energy Conversion

**DOI:** 10.1002/advs.202000286

**Published:** 2020-04-28

**Authors:** Zhenkun Zhang, Wenhao Shen, Lingxin Lin, Mao Wang, Ning Li, Zhifeng Zheng, Feng Liu, Liuxuan Cao

**Affiliations:** ^1^ College of Energy Xiamen University Xiamen Fujian 361005 P. R. China; ^2^ State Key Laboratory of Nuclear Physics and Technology Peking University Beijing 100871 P. R. China; ^3^ Center for Quantitative Biology Peking University Beijing 100871 P. R. China

**Keywords:** energy conversion, graphene oxide, high ion permeability, ion selectivity, vertically transported

## Abstract

Reverse electrodialysis is a promising method to harvest the osmotic energy stored between seawater and freshwater, but it has been a long‐standing challenge to fabricate permselective membranes with the power density surpassing the industry benchmark of 5.0 W m^−2^ for half a century. Herein, a vertically transported graphene oxide (V‐GO) with the combination of high ion selectivity and ultrafast ion permeation is reported, whose permeation is three orders of magnitude higher than the extensively studied horizontally transported GO (H‐GO). By mixing artificial seawater and river water, an unprecedented high output power density of 10.6 W m^−2^ is obtained, outperforming all existing materials. Molecular dynamics (MD) simulations reveal the mechanism of the ultrafast transport in V‐GO results from the quick entering of ions and the large accessible area as well as the apparent short diffusion paths in V‐GO. These results will facilitate the practical application of osmotic energy and bring an innovative design strategy for various systems involving ultrafast transport, such as filtration and catalysis.

## Graphical abstract

Vertically transported graphene oxide is prepared to extract osmotic energy from mixing artificial seawater and river water with an unprecedentedly high power density of 10.6 W m^−2^. It surpasses the industry benchmark of 5.0 W m^−2^ and outperforms all the reported nanoporous membranes. Under more optimized conditions, the power density can be further enhanced to be 34 W m^−2^.

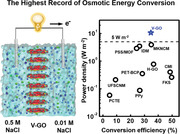

## 1 Introduction

To address the challenge of global warming and environmental deterioration, renewable energies have become an urgent demand for the sustainable development of human society.^[^[qv: ^1,2^]^]^ Osmotic energy stored as the form of salinity difference between seawater and freshwater, is a completely clean energy source without any pollution and carbon dioxides emission.^[^[qv: ^3–5^]^]^ Reverse electrodialysis, a conventional method used to retrieve salinity gradient energy, is constrained by the poor performance of permselective membranes during the past half a century.^[^[qv: ^6^]^]^ Inspired by the biological ion channels, artificial nanopores are used for osmotic energy conversion owing to the exceptional ion transport properties on nanoscale.^[^[qv: ^7^]^]^ The nanofluidic reverse electrodialysis system (NREDS) was realized on many kinds of single pore model,^[^[qv: ^8–10^]^]^ indicating great potential superior to conventional commercial materials. Toward practical application, the NREDS were widely studied in a variety of porous materials, including polymeric membranes,^[^[qv: ^11,12^]^]^ inorganic carbon materials,^[^[qv: ^13,14^]^]^ silicon‐based materials,^[^[qv: ^15^]^]^ aluminum oxide (AAO) template,^[^[qv: ^16^]^]^ compound materials,^[^[qv: ^17–19^]^]^ and stacked 2D materials.^[^[qv: ^20,21^]^]^ Up to now, the highest record by mixing seawater and river water is 4.1 W m^−2^ obtained in MXene/Kevlar nanofiber composite membranes.^[^[qv: ^22^]^]^ Although it largely outperforms commercial ion exchange membranes by nearly an order of magnitude, the power density is still below the commercialization benchmark of 5.0 W m^−2^ in the seawater/fresh water systems.^[^[qv: ^23,24^]^]^

From the aspects of fundamental transport mechanism, the essential approach to promote the performance of nanoporous membrane is to break through the trade‐off between the ion permeability and selectivity.^[^[qv: ^25–27^]^]^ The recent rise of 2D materials provide an encouraging solution as the fast ion transport was observed in the interstitial space between restacked 2D nanosheets combined with high ion selectivity.^[^[qv: ^28^]^]^ More interestingly, further researches reveal the unidirectional interlayer paths of vertically transported 2D nano‐sheet structure enable an ultrafast migration of ions extremely superior to the counterpart with horizontally stacked structure. This exceptional character has been employed to fabricate high‐performance supercapacitors and electrodes with rapid electrolyte ion diffusion, high areal capacitance and fast response.^[^[qv: ^29–31^]^]^ It can be expected that if the 2D membrane allows vertical‐transportation of ions (ions permeate along the lamination direction), it should have outstanding ion permeability performance. However, the vertically transported 2D membrane is still absent in osmotic power generation but urgently demanded from either mechanism research or practical application.

Herein, we report a vertically transported graphene oxide (V‐GO) prepared through vacuum filtration combined with MEMS fabrication technology. Benefit from the unidirectional lamellas, the ion has ultrafast ion permeation in V‐GO, with three orders of magnitude higher than that in horizontally transported GO (H‐GO). Due to the extraordinary combination of selectivity and permeability, the V‐GO can achieve an unprecedented output power density of 10.6 W m^−2^ by mixing seawater and river water, which largely outperforms the existing materials and beyond the critical value of 5.0 W m^−2^ for industrial development requirement. The theoretical analysis and molecular dynamics simulations reveal the ultrafast ion transport mechanism in V‐GO. Besides the apparent short diffusion paths in unidirectional lamellas, the other two factors, the loading time of ions entering GO without moving back and the accessible area on the membrane surface, play more essential roles in the ultrafast ion transport in V‐GO. These findings can greatly promote the practical applications of osmotic energy and open an innovative avenue toward various systems that involve ultrafast transport, such as filtration and catalysis.

## 2 Results and Discussion

### 2.1 Fabrication and Characterization of GO

**Figure 1:**
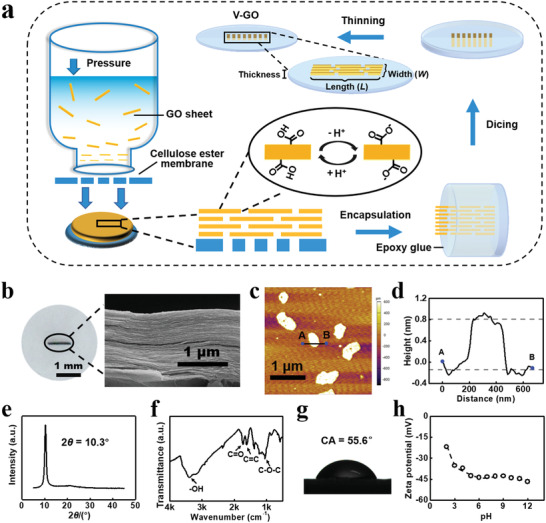
Preparation and characterization of V‐GO. a) The fabrication process of V‐GO. b) SEM image of V‐GO showing compactly packed lamellar structures. c,d) A typical AFM observation of the GO sheets suggests that the thickness of individual GO sheets is about 0.9 ± 0.1 nm. e) Sharp XRD peak at 10.3° indicates the uniform interlayer distance of 0.86 nm. f) FT‐IR spectra indicates the chemical functional groups in synthetic GO sheets. g) The GO are hydrophilic with surface contact angles of 55.6°. h) Surface charge properties of GO colloids under varied pH conditions (0.1 mg mL^−1^).

**Figure** **1**a shows the fabrication processes for V‐GO. First, GO membranes were formed through vacuum filtration. They were further cut into proper pieces and encapsulated by epoxy glue. The method of mechanical dicing, polishing, and ion thinning were used to make the vertically transported GO structures exposed from the cured epoxy glue and to reduce the membrane thickness. In this way, we can obtain the V‐GO with appropriate thickness and uniform lamellar microstructures (Figure 1b). The atomic force microscope (AFM) observation suggests that the lateral size distribution of these GO sheets ranges from 400 to 1000 nm (Figure 1c; Figure S1, Supporting Information) and the average height is 0.9 ± 0.1 nm (Figure 1d; Figure S2, Supporting Information). X‐ray diffraction (XRD) patterns of (001) plane indicate that the interlayer spacing of the GO is 0.86 nm (Figure 1e). The interlayer spacing is larger than that of pristine graphite, suggesting the oxygen‐containing functional groups were successfully introduced on the surface of the nanosheet during the process of preparation.^[^[qv: ^32^]^]^ Fourier transform infrared spectroscopy (FT‐IR) shows the presence of multiple oxygen‐containing functional groups in graphene oxide (Figure 1f). These oxygen‐containing functional groups can be quantitatively analyzed by X‐ray photoelectron spectroscopy (XPS),^[^[qv: ^33^]^]^ in which the carboxyl acid group is 1.2% of the total carbon content (Figures S3 and S4, Supporting Information). The surface charge density of GO sheet is −73.8 mC m^−2^ after the complete ionization of carboxyl acid group, which is in agreement with the previous results.^[^[qv: ^20^]^]^ The GO are hydrophilic with surface contact angles of 55.6° (Figure 1g). The zeta potential of the GO aqueous solution pulverized by ultrasonication shows the stability over the pH range of 3–11 (Figure 1h).^[^[qv: ^34^]^]^ These characterization results are consistent with previous literature reports.^[^[qv: ^20,33–36^]^]^

### 2.2 Ultrahigh Output Power Density of V‐GO

**Figure 2:**
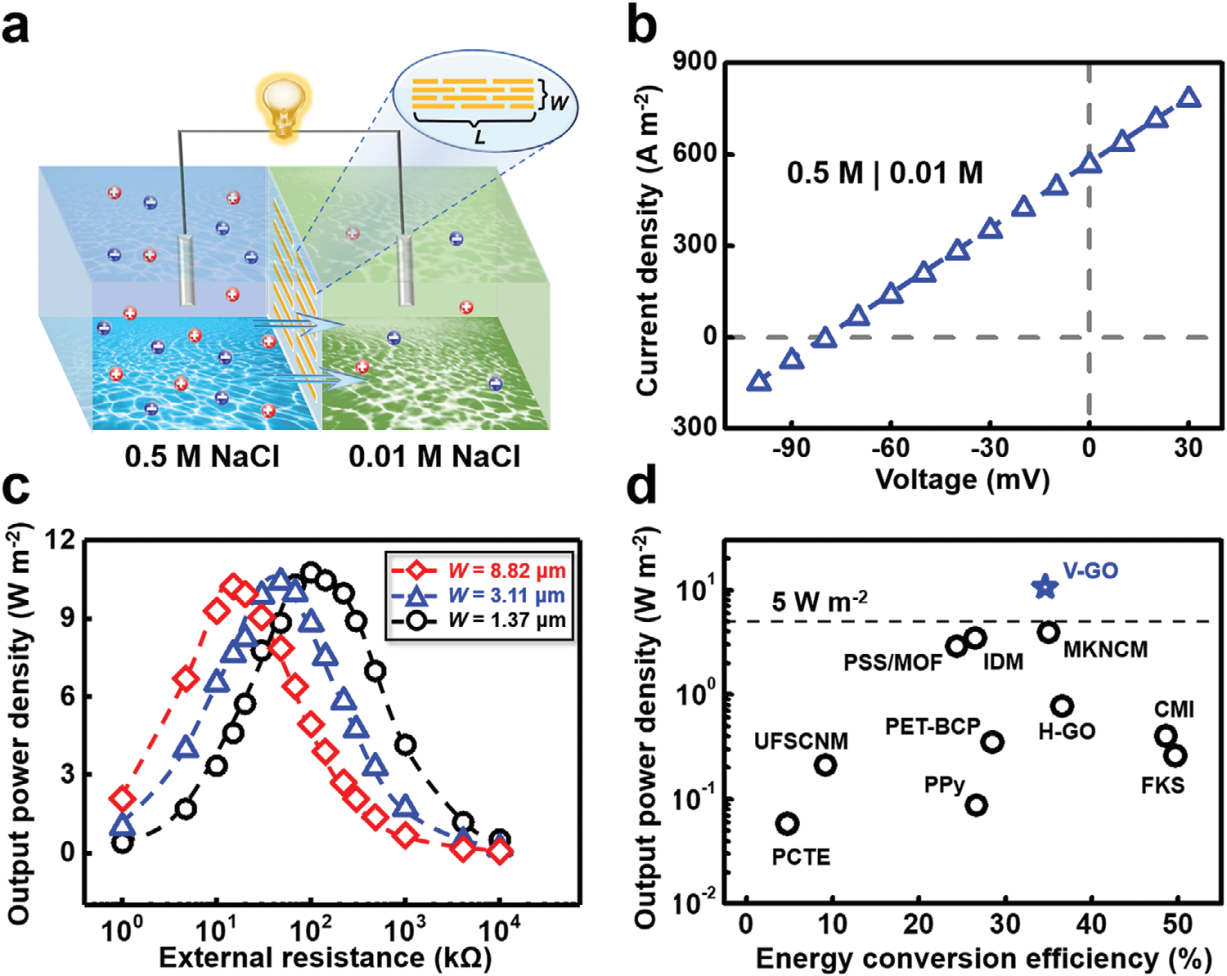
Ultrahigh output power density of V‐GO. a) Schematic of energy harvesting from the seawater and river water. b) IV curve of V‐GO. c) The output power measured in external loads. The obtained output power density of V‐GO is as high as 10.6 W m^−2^. d) Benchmark of the V‐GO with other reported osmotic energy conversion systems for the output power density and energy conversion efficiency. The V‐GO has the highest output power density.

We measured the output power of V‐GO to harvest the osmotic energy stored in the artificial seawater and river water (**Figure** **2**a). The V‐GO were placed between 0.5 m NaCl and 10 × 10^−3^ m NaCl, which is common used in the previous researches to simulate the seawater and river water.^[^[qv: ^5,37^]^]^ Electric power could be generated from the osmotic energy because of the charge separation in the ion‐selective nanochannels.^[^[qv: ^38^]^]^ The membrane potential of 79.1 mV and diffusion current density of 570 A m^−2^ were respectively read from the intercepts on the horizontal and longitudinal coordinate (Figure 2b). Reference electrodes were applied in electrical measurement to eliminate the contribution of redox potential generated by the unequal potential change at the electrode–solution interface.^[^[qv: ^10,22^]^]^

The harvested electric power can be output to an external circuit (Figure 2c). Through measuring the electrical signals in the electronic load, the electric power (*P*_R_) consumed on the external resistance (*R*) can be directly calculated by *P*_R_ = *I* ^2^ × *R*. The current decreases gradually with the increment of external resistance (Figure S5, Supporting Information). And the output power achieves its peak value when the load resistance is equal to the internal resistance of membrane. As the lengths of V‐GO are fixed to about 1120 µm and widths (*W*) are 1.37, 3.11, and 8.82 µm, the output powers reach the maximum when the external resistance is about 100, 45, and 15 kΩ, respectively. The membrane thickness of V‐GO is about 350 µm.

The obtained output power density of V‐GO can achieve an unprecedented high value of 10.6 W m^−2^, which is the highest reported record among all existing materials used to harvest osmotic energy, including PCTE,^[^[qv: ^39^]^]^ UFSCNM,^[^[qv: ^17^]^]^ PSS/MOF,^[^[qv: ^18^]^]^ IDM,^[^[qv: ^13^]^]^ PPy,^[^[qv: ^12^]^]^ PET‐BCP,^[^[qv: ^19^]^]^ MKNCM,^[^[qv: ^22^]^]^ H‐GOMs,^[^[qv: ^20^]^]^ CMI,^[^[qv: ^13^]^]^ and FKS (Figure 2d; Table S1, Supporting Information).^[^[qv: ^13^]^]^ Most importantly, this extremely high output power density apparently exceeds the commercialization benchmark of 5.0 W m^−2^ for the first time, which probably promotes the industrialized utilization of salt difference energy.^[^[qv: ^5^]^]^ To fairly reflect the properties of materials, the data presented in Figure 2d are all under the same concentration difference of 500 × 10^−3^ m | 10 × 10^−3^ m NaCl, close to the concentration condition of most river water and seawater.^[^[qv: ^20,22^]^]^ The characterization of the geometic size of V‐GO samples are listed in Figures S6–S14 in the Supporting Information.

### 2.3 Enhancement of the Power Density of V‐GO

**Figure 3:**
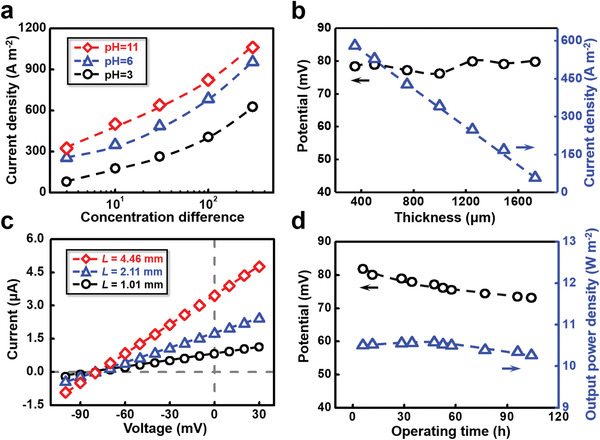
Energy conversion of V‐GO in different conditions. a) The diffusion current density increases with the applied concentration difference and pH value. b) The generated current density declines with the increasing membrane thickness, showing Ohm‐like dependence. The membrane potential is not sensitive to the membrane thickness. c) The diffusion current increases with the membrane length (*L*), but the membrane potential remains stable. d) V‐GO can keep stable performance in electrolyte solution for several days. The applied concentration difference is 0.5 m | 0.01 m NaCl.

The power generation performance of V‐GO can be further improved through the increment of concentration difference and pH value of electrolyte solution (**Figure** **3**a; Figure S15, Supporting Information), which is consistent with the conventional viewpoint.^[^[qv: ^40^]^]^ Under more optimized conditions (1 m | 1 × 10^−3^ m KCl, pH = 11), the power density of V‐GO can be further enhanced to be 34 W m^−2^, which is significantly greater than the recent record of 21 W m^−2^ under the same condition using MXene membranes.^[^[qv: ^41^]^]^ Actually, the osmotic energy conversion of V‐GO can outperform all reported materials in testing conditions other than the standard artificial seawater and river water (Table S2, Supporting Information).

Besides, the output power density of V‐GO can also be significantly enhanced through reducing the membrane thickness. As shown in Figure 3b, the power generation shows classical Ohm‐type membrane‐thickness dependence: the diffusion current density linearly decreases with the reduction of membrane thickness from 1800 to 350 µm; the membrane potential keep constant at about 80 mV with the change of membrane thickness. This is easy to understand because the membrane thickness only affects transmembrane resistance instead of the charge selectivity.^[^[qv: ^42^]^]^ Accordingly, the total power density grows lineally with the thinning of V‐GO (Figure S16, Supporting Information). Of note, the smallest thickness of V‐GO in this work is about 350 µm owing to the limitation of our current preparation technology. Even in such thick membrane compared to the existing materials,^[^[qv: ^17,22,43^]^]^ the output power density obtained in 350 µm‐thick V‐GO is higher than 10 W m^−2^. If the membrane thickness were reduced to below 30 µm, the power generation of V‐GO has nearly ten times growth potential since the previous research has pointed out that the power density could be linearly enhanced within this thickness range.^[^[qv: ^42^]^]^

### 2.4 Scalability and Aqueous Stability

The total output power of V‐GO can be easily promoted by enlarging the effective membrane area. As shown in Figure 3c, the diffusion current is facilitated by increasing lengths (*L*) from 1.01 to 4.46 mm. As expected, the V‐GO provides stable membrane potential of about 80 mV because of the constant ion selectivity. Similarly, the prolonged width (*W*) also raises the diffusion current linearly (Figure S17, Supporting Information). In fact, the output electric powers show an excellent linear relationship with respect to the testing area of V‐GO while the power density keeps constant (**Table** **1**, Supporting Information). This result suggests that the total output power of V‐GO has the potential to scale up. The next challenge is to improve the fabrication method to produce V‐GO in large‐areas using high throughput manufacturing routes, which is still a widely recognized hurdle for promoting the practical application of all the emerging 2D materials.^[^[qv: ^4,44^]^]^

**Table 1:** The output power of different V‐GO samples. The applied solution condition is 0.5 m | 1 × 10^−2^ m NaCl. The membrane thickness is ≈350 µm. Errors of the length and width are the standard deviation of multiple measurements. Errors of the output power density are calculated based on error propagation

Sample	Length [mm]	Width [µm]	Current [µA]	Membrane potential [mV]	Output power density [W m^−2^]
1	1.016 ± 0.001	1.47 ± 0.15	0.816	79.6	10.87 ± 0.74
2	2.107 ± 0.002	1.51 ± 0.17	1.72	78.6	10.62 ± 0.38
3	4.462 ± 0.003	1.42 ± 0.18	3.45	79.1	10.77 ± 0.21
4	1.064 ± 0.001	0.86 ± 0.08	0.505	80.2	11.07 ± 1.12
5	0.989 ± 0.001	1.37 ± 0.15	0.804	79.3	11.76 ± 0.95
6	0.963 ± 0.001	3.15 ± 0.19	1.76	78.4	11.37 ± 0.23
7	1.043 ± 0.002	6.08 ± 0.32	3.45	80.4	10.94 ± 0.09
8	1.026 ± 0.002	8.92 ± 0.48	5.23	77.8	11.12 ± 0. 07

The V‐GO also possesses the excellent stability in aqueous solution environment as well as the high power density. The measure of electrical power generation lasted for more than 100 h. The initial membrane potential is 83 mV, and the magnitude of voltage drop can be controlled within 10% during the 100‐hour test. Meanwhile, the maximum output power density is basically maintained above 10.5 W m^−2^ (Figure 3d), suggesting the satisfying stability in practical application.

### 2.5 High Ionic Permeability and Selectivity

**Figure 4:**
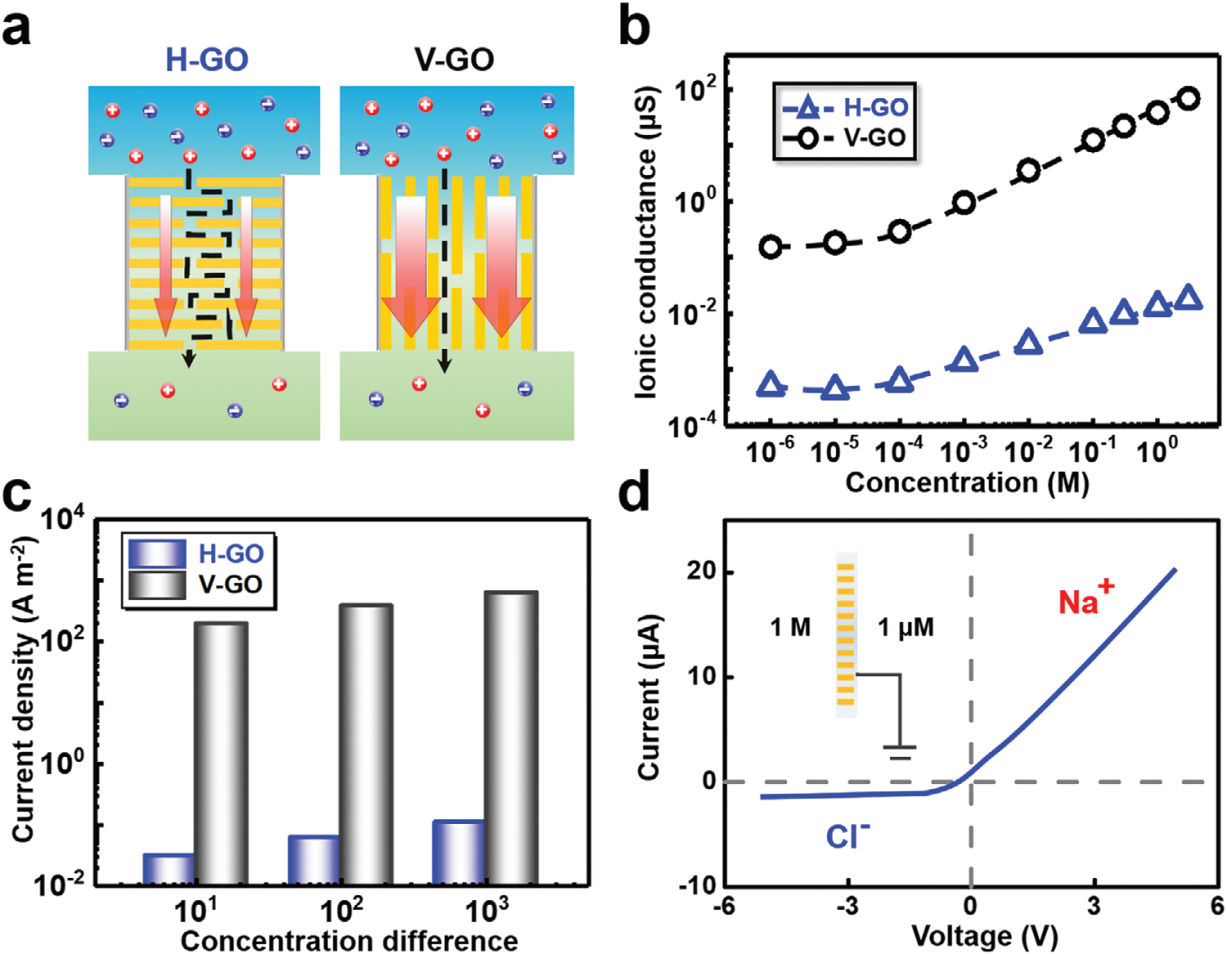
High permeability and selectivity of V‐GO. a) Schematic of ion transport in H‐GO and V‐GO. b) Ionic conductance of V‐GO is several hundred higher than that of H‐GO. The effective area of H‐GO and V‐GO is 200 × 200 µm and 1140 × 3.11 µm, respectively. c) Current density of V‐GO driven by ion concentration gradient is more than that of H‐GO with three orders of magnitude. The electrolyte solution is NaCl with low concentration side of 1 × 10^−3^ m. d) Ion selectivity of the V‐GO is verified by the ionic current under asymmetric concentration. The cation transference number (*t_+_*) is 0.916.

To understand the origin of the high performance of V‐GO in osmotic energy conversion, we systematically investigate ion selectivity and permeability, the two most important factors affecting the output power, in V‐GO with the extensively used H‐GO as a comparison.^[^[qv: ^33,45^]^]^ We prepared V‐GO and H‐GO with the same fabrication condition to ensure they have the identical interlayer spacing and chemical composition, which accounts for the similar ion selectivity in V‐GO and H‐GO.^[^[qv: ^46^]^]^ However, the different orientations of 2D flakes induce two types of ion transport modes across the membranes (**Figure** **4**a). As shown in Figure 4b, the ionic conductance presents the electric‐field‐induced ion transportation with the electrolyte concentration ranged from 1 × 10^−3^ to 0.1 × 10^−3^ m, and the strong surface‐charge‐governed ion transporting behavior when the ion concentration further decreases to lower than 0.1 × 10^−3^ m, which is consistent with those reported in the literatures.^[^[qv: ^20,47^]^]^ Intriguingly, the ionic conductance in V‐GO is several hundred times higher than that in H‐GO in all testing concentration conditions, which is consistent with the data plotted in Figure 2d. The effective testing areas of H‐GO and V‐GO are 200 µm × 200 µm and 1.142 mm × 3.11 µm, respectively. The data plotted in Figure 4b are the result of normalization of testing area. Besides, the diffusion current density in V‐GO driven by the salinity gradient also extremely surpasses those in H‐GO, which is nearly 5000 times enhanced in V‐GO (Figure 4c). V‐GO also allows the ultrafast transport of divalent cation (Figure S18, Supporting Information). It also can achieve high performance in more kinds of electrolyte, including LiCl, NaCl, and KCl (Figure S19, Supporting Information). The results also indicate that higher mobility of cations can effectively enhance the diffusion current and membrane potential, consistent with the existing literature.^[^[qv: ^40^]^]^

We further analyzed the ion selectivity of V‐GO through measuring the IV curve in NaCl solutions with highly different concentrations (1 m | 1 × 10^−3^ m). Because of the imbalanced electrolyte solution in the two sides of the membrane, the major ionic carriers across the membrane come from the left reservoir. Thus, the positive and negative ionic currents are dominated by Na^+^ and Cl^−^, respectively. As shown in Figure 4d, the tested Na^+^ current is 10.9 times higher than the Cl^−^ current. Based on the formula of *t_+_* = *I_+_*/(|*I_+_*| + |*I_−_*|), the cation transference number (*t_+_*) of the V‐GO is 0.916,^[^[qv: ^48^]^]^ which is almost the same as H‐GO (0.909), indicating strong cation selectivity in both V‐GO and H‐GO (Figure S20, Supporting Information).

### 2.6 Ultrafast Transport Mechanism

**Figure 5:**
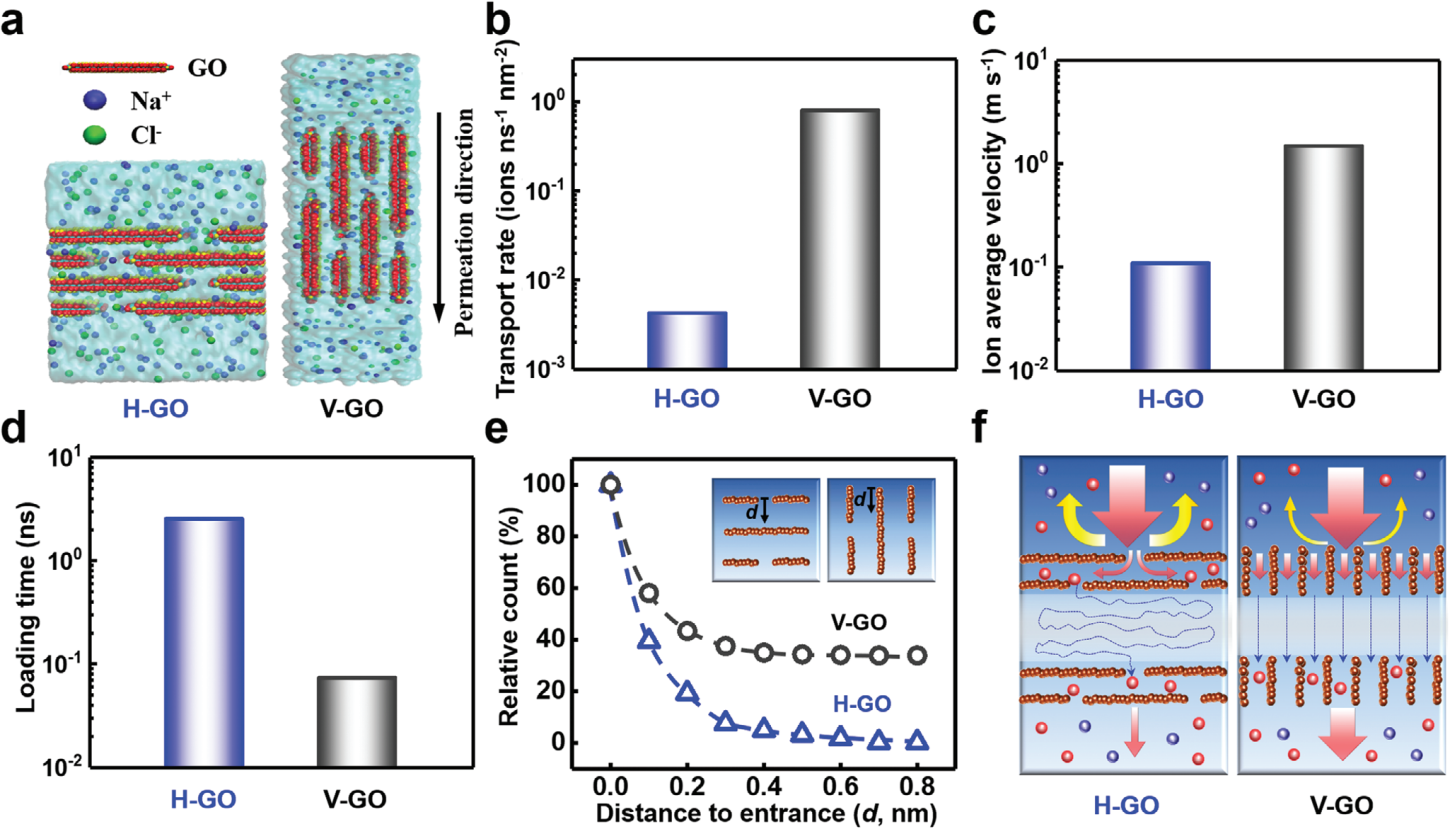
MD simulations of ionic transport through H‐GO and V‐GO. a) The MD simulation model of the H‐GO (left) or V‐GO (right) is consisted of four GO flakes with the interlayer spacing of 1 nm. b) The transport rate of Na^+^ ions through V‐GO is 190 times larger than that of H‐GO. c) The zigzag trajectories in H‐GO remarkably slow down the average velocity of ions, which is more than 13.7 times less than that in V‐GO. d) The loading time in H‐GO is more than 30 times longer than that in V‐GO. e) For individual orifice, the relative ion number in H‐GO decreases with the distance from the entrance much more drastically than that in V‐GO. f) Schematic of the ultrafast ionic transport mechanism through V‐GO superior to H‐GO.

To reveal the origin of ultrafast ion permeation in V‐GO, we performed MD simulations to explore the mechanism of ionic migration through V‐GO and H‐GO.^[^[qv: ^49^]^]^ We constructed an atomic model of the GO with the interlayer spacing of 1 nm and surface charge density and elemental composition similar to experimental samples (**Figure** **5**a; Figure S21, Supporting Information). The MD simulations show that the transport rate of Na^+^ ions through V‐GO normalized by the surface area is 190 times larger than that of H‐GO (Figure 5b). It is easily thought to stem from the different passing paths inside V‐GO and H‐GO. During the ions passing through H‐GO, they have to take zigzag trajectories to go through the gaps between adjacent layers (Figure S22a, Supporting Information). In sharp contrast, most of the ions can pass straight through a single channel inside V‐GO (Figure S22b, Supporting Information). As a result, the ion average velocity along the permeation direction across H‐GO remarkably slows down, which is more than 13.7 times less than that in V‐GO (Figure 5c).

Nevertheless, the differences in passing paths are not adequate to account for the disparities in permeability by more than two orders of magnitude. Another crucial factor responsible for the ultrahigh ionic permeation is identified as the rapid access provided by the unique geometric structure in V‐GO. The access process of ions from the outer region into the channel can be evaluated as the loading time,^[^[qv: ^50,51^]^]^ which is the time elapse between two ions subsequently entering GO without moving out. The MD simulation illustrates that the loading time in H‐GO is 34.8 times longer than that in V‐GO (Figure 5d). There are two main factors contributing to this difference. First, Na^+^ ions are more difficult to go inside H‐GO than V‐GO. During the entrance of Na^+^ ions into H‐GO, the tortuous geometric structures produce a strong barrier to impede the access of Na^+^. For individual orifice, the relative ion numbers in H‐GO decrease with the distance from the entrance much more drastically than that in V‐GO (Figure 5e). In the 0.8 nm depth from the orifices, there are nearly 33.8% ions staying inside V‐GO. In contrast, only 0.2% ions remain in H‐GO at the same depth. It results from the evident ion enrichment at the entrance of H‐GO (Figure S23, Supporting Information), which accordingly induces strong electrostatic repulsion and dehydration barrier for the Na^+^ ions to enter. These huge disparities suggest the single channel in V‐GO has higher transport efficiency rather than the counterpart in H‐GO. Second, V‐GO offer a much greater accessible area for ions to enter compared with H‐GO. In the experiment, the proportion of entrance area to total surface area in V‐GO can achieve to amazingly about 50%, which largely surpasses that in H‐GO (<1%). It is worthy to point out that, due to the limitation of computational capacity, the proportion of the entrance area of H‐GO in the simulation is much larger than that in the experiment. Hence the transport ratio between V‐GO and H‐GO is underestimated in the simulation compared with the experiment.

## 3 Conclusion

In conclusion, we present V‐GO through the vacuum filtration and MEMS fabrication technology. Owing to the extraordinary combination of ion selectivity and permeability, the V‐GO can achieve an extremely high output power density of 10.6 W m^−2^ from artificial seawater and river water, which apparently outperforms the existing materials and beyond the critical value of 5.0 W m^−2^ for industrial development requirement. The molecular dynamics simulations reveal the mechanism of ultrafast ion transport in V‐GO (Figure 5f). The penetrating passages in V‐GO allow a short migration distance and efficiently promote the ionic transport rate. Furthermore, the unique geometric structures in V‐GO lower the entrance barrier and provide the rapid and efficient access to the inside channel. These findings can bring innovative design strategy for various ion transport systems, including catalysis, chemical sensing, ion filtration, water purification and energy conversion.

## 4 Experimental Section

##### Fabrication

GO sheets stacked from bottom to top onto the supporting membrane by vacuum filtration. Afterward, the GO membranes were dried in air at room temperature to remove residual water. Then, they were cut into proper pieces and encapsulated by epoxy glue. The method of mechanical dicing, polishing and ion thinning were used to make the vertically transported GO structures exposed from the cured epoxy glue and to reduce the membrane thickness. In this way, the V‐GO could be obtained with appropriate thickness and uniform lamellar microstructures. The detailed procedure is described in the Supporting Information.

##### Characterization

The zeta potential and size distribution of GO colloids (0.1 mg mL^−1^) were measured with Malvern Zetasizer NanoZS90. The size and thickness of GO sheets were characterized by atomic force microscope (FM‐Nanoview 6800AFM). The interlayer distance was tested on a polycrystalline X‐ray diffractometer with a Cu K*α* radiation source (Rigaku Ultima IV). Fourier transform infrared spectrometer (Nicolet Is5) was employed to map the distribution of chemical bond on the surface of GO sheets. The hydrophilicity of GO was characterized by contact angle measuring instrument (JC200JC1). The microstructure was observed by the field emission scanning electron microscope (SUPRA 55 SAPPHIRE).

##### MD Simulations

An atomic GO model was constructed based on the characterization parameters of the experimental samples (Supporting Information).^[^[qv: ^52^]^]^ All MD simulations were performed using GROMACS4.6 with CHARMM36 force field and TIP3P water model. The simulation used Van der Waals interactions with a cutoff of 1 nm and Particle‐Mesh Edward electrostatics. Periodic boundary conditions were applied to all three directions. The temperature was maintained at 300 K using v‐rescale. Each ionic transport simulation was run for 40 ns with the constant number of particles, volume, and temperature (NVT) ensemble after the solvation reaches equilibrium. Only later 35 ns of the simulation were used for data analysis to ensure that the ion transport reaches equilibrium.

## Conflict of Interest

The authors declare no conflict of interest.

## Supporting information

Supporting Information
