# Supplementary material for: Vertically Transported Graphene Oxide for High‐Performance Osmotic Energy Conversion
Source: Adv Sci (Weinh). 2020 Apr 28;7(12):2000286. doi: 10.1002/advs.202000286 (PMC7312320; doi:10.1002/advs.202000286)
Supplement: Supplementary file 1 — Supporting Information [file ADVS-7-2000286-s001.pdf]

Copyright WILEY-VCH Verlag GmbH & Co. KGaA, 69469 Weinheim, Germany, 2020.

Supporting Information

Vertically-Transported Graphene Oxide for High-Performance Osmotic Energy Conversion

Zhenkun Zhang, Wenhao Shen, Lingxin Lin, Mao Wang, Ning Li, Zhifeng Zheng, Feng Liu, and Liuxuan Cao**

Z. Zhang, L. Lin, Prof. N. Li, Prof. Z. Zheng, Prof. L. Cao
College of Energy, Xiamen University, Xiamen, Fujian 361005, P. R. China.
E-mail: caoliuxuan@xmu.edu.cn

W. Shen, M. Wang, Prof. Feng Liu.
State Key Laboratory of Nuclear Physics and Technology, Peking University, 100871 Beijing, P. R. China.
Center for Quantitative Biology, Peking University, 100871 Beijing, P. R. China.
E-mail: liufeng-phy@pku.edu.cn

Table of contents:

1. Fabrication of V-GO.

2. Characterization of V-GO.

3. Output power in V-GO.

4. Geometic size measurments of V-GO samples.

5. Electrical Measurement.

6. Energy conversion efficiency.

7. Energy conversion under different electrolyte conditions.

8. Membrane thickness dependent energy conversion.

9. Membrane area dependent energy conversion.

10. Ion selectivity and permeation.

11. MD simulation.

1. Fabrication of V-GO.

Graphene oxide (GO) was synthesized from purified natural graphite by the modified Hummers' method.[1,2] Graphite powder (98.5%, 8 g), sodium nitrate ($NaNO_3$, 99.0%, 8 g) and concentrated sulfuric acid (H_2SO_4, 98%, 384 ml) were completely mixed at 263.2 K for 12 hours. Then potassium permanganate ($KMnO_4$, 99.0%, 48 g) was added slowly to the mixture at 263.2 K. The mixture was firstly sufficiently stirred at 273.2 K for 1 hour, and then was reacting at 308.2 K for 12 hours. While maintaining the stirring rate and fixing temperature at 273.2 K, deionized water (1000 ml) was added slowly to the mixture (40 min) followed by the addition of hydrogen peroxide (H_2O_2, 99.0%, 10 ml, 100 min). Afterwards, the dispersion solution was treated by centrifugal process at 12,000 rpm for 20 min several times until the pH of the supernatant was approximately equal to 7. Finally, the resulting GO dispersion was freeze-dried to obtain fluffy graphene oxide (GO) and stored in dark.

GO and deionized water with a mass ratio of 1:1000 changed into dispersion by ultrasonic cell pulverizer with the power of 10 W. The dispersion was further isolated by centrifuging at 9,000 rpm for 6 min to remove the lager and insoluble GO sheets. GO dispersion (～1 mg mL^{-1}) was filtrated through a cellulose ester membrane (47 mm in diameter, effective pore size of 200 nm) by the method of vacuuming.[2,3] Afterwards, the GO were dried in air at room temperature to remove residual water. Through this process, the horizontally stacked GO (H-GO) were obtained. To prepare V-GO, the H-GO were cut into appropriate pieces and encapsulated by epoxy glue. Exposure to ultraviolet light for 2 minutes made the epoxy glue completely cured. Through mechanical dicing, the glue formed a membrane with the thickness of about 1 mm. In addition, the membrane thickness was further reduced to 350 μm by polishing. Meanwhile, the vertically-transported GO structures were exposed from the cured epoxy glue. Finally, the ion thinning was employed to smooth the surface of the V-GO.

2. Characterization of V-GO.

A drop of diluted GO dispersion (0.5 μg ml^{-1}) was deposited on the flat surface of a mica slice and was observed by AFM (FM-Nanoview 6800AFM). The AFM observation suggests that the average thickness of GO sheets was about 0.9±0.1 nm and the lateral size distribution of these GO sheets ranged from 400 nm to 1000 nm. The lateral size distributed of the GO sheets was confirmed to be between 400 nm and 1000 nm at the concentration of 0.1 mg mL^{-1} with Malvern Zetasizer NanoZS90 (Figure S1, Supporting Information).

The interlayer spacing of dried GO was tested on a polycrystalline X-ray diffractometer with a Cu Kα radiation source (Rigaku Ultima IV). X-ray diffraction (XRD) patterns showed

the sharp peak at 10.3° with the scanning speed of 1° min^{-1}. It corresponds to the interlayer spacing of 0.86 nm.

Energy Dispersive Spectrometer (EDS) was used to analyze the types of elements in the synthesized graphene oxide. Only the elements of carbon and oxygen were observed in the EDS spectra, with the contents of 62.5% and 37.5%, respectively (Figure S3, Supporting Information). It showed that the graphite was partially oxidized.

Fourier transform infrared spectroscopy (FT-IR) shows the presence of multiple oxygen-containing functional groups in graphene oxide. The characteristic GO peaks were observed at 3350, 1730, 1618, 1360, and 1050 cm^{-1}, which correspond to the stretching vibration of -OH, C=O, C=C, and C-O-C groups, respectively.[2] Among them, the presence of carboxyl group makes the GO to be cation selective.

The content of these oxygen groups can be quantitatively analyzed by X-ray photoelectron spectroscopy (XPS).[2,4] A typical XPS pattern on GO is shown in Figure S4. The total spectra (solid line) can be decomposed to four Lorentzian peaks corresponding to C=C with binding energy of 284.8 eV, C-O with binding energy of 286.7 eV, C=O with binding energy of 287.6 eV, and O=C-OH with binding energy of 289.2 eV. According to the intensity of the O=C-OH peak, the numerical density of the carboxylic acid on GO sheets can be calculated to be about 1.2% in the total carbon content. The carbon-carbon bond length in GO sheets is 0.14 nm, equivalent to 39 carbon atoms in 1 nm^2 area. Then, the numerical density of the carboxylic acid groups is estimated to be 0.46 nm^{-2}, equivalent to a surface charge density of -73.8 mC m^{-2}, which is in agreement with the previous work.[5]

3. Output power in V-GO.

By mixing concentrated (500 m M) and diluted (10 m M) NaCl solution, the osmotic energy is converted to electric power through the charge separation in the ion selective nanoporous membrane. The harvested electric power can be output to an external circuit. As shown in Figure S5, the current densities measured in external load all decreased with the load resistance. But the output power achieves its peak value when the load resistance is equal to the internal resistance of membrane. For the V-GO with the varied areas of 1.37 µm × 0.981 mm, 3.11 µm × 0.963 mm and 8.82 µm × 1.026 mm, the output powers reach the maximum when the external resistance is about 100 kΩ, 47 kΩ and 15 kΩ, respectively.

4. Geometic size measurments of V-GO samples.

The effective area and thickness of the V-GO were characterized by scanning electron microscope (SEM). The samples were made into SEM samples after electrical measurement. With Au deposition, the internal uniform lamellar microstructures in V-GO could be observed. Based on the SEM (SUPRA 55 SAPPHIRE) observation, the cross-section of the V-GO samples is rectangular. Considering that the width (*W*) is not uniform, 100 data of the width were measured throughout the whole sample. Then the average values and standard deviation were calculated from all measured values. The width from different V-GO, is 1.37 ± 0.17 μm (Figure S6, Supporting Information), 3.11 ± 0.19 μm (Figure S7, Supporting Information), and 8.82 ± 0.48 μm (Figure S8, Supporting Information). The width uniformity of different samples is plotted in Figure S9. The samples were cut in the middle of the cross-section to expose the dimension of the thickness. The thickness was measured through a similar method. The typical SEM image, the thickness uniformity, and the average thickness of several samples are presented in Figure S10-S13. The effective lengths (*L*) of different V-GO samples were obtained by averaging the results of multiple measurements. Figure S14 shows the effective lengths (*L*) of different V-GO samples are 1.026±0.002 mm, 2.107±0.007 mm, and 4.447±0.016 mm.

5. Electrical Measurement.

The membrane samples were mounted between two electrolyte cells. Each cell was filled with 10 mL NaCl solution. Varied concentrations of NaCl solution were sequentially applied at the two sides of the nanopore, constructing the concentration gradient. The IV curves of the V-GO system were recorded by a source meter (Keithley 6487). The degassed Milli-Q water (18.2 MΩ·cm) was used to prepare all testing solutions. To evaluate the effect of electrode potential on electrical measurement, the reference electrodes were used to apply the transmembrane potential and measure the resulting current-voltage responses of the testing V-GO.[6] The intercept on the vertical axis (I_{sc}) represents the net diffusion current. Correspondingly, the intercept on the horizontal axis (U_{oc}) represents the membrane potential.

6. Energy conversion efficiency.

Ion concentration difference is one type of Gibbs free energy that can be converted to electric power. Considering the two bulk reservoirs connected with a series of nanopores with surface charge, spontaneous ion diffusion along the concentration gradient from the high-concentration solution (C_H) to the low-concentration solution (C_L) results in the mixing of

electrolyte solution. In an infinitesimal time unit (*dt*), the Gibbs free energy loss can be described as,

$$dG = -\frac{RT}{F}\ln\frac{a_H}{a_L}(|I_+| + |I_-|)\,dt \tag{S1}$$

where $\alpha_{H/L}$ is the chemical activity of the ionic species on C_H or C_L side; I_+ and I_- is the ion flux contributed by cations and anions; F, R, T are the Faraday constant, the universal gas constant, and the temperature, respectively. In the diffusion process, the charge selectivity of nanopore causes asymmetric migration of cations and anions which can convert the Gibbs free energy partly into electric work (*dW*) in the form of net diffusion current (I_{net}) and transmembrane potential (ε),

$$dW = I_{net}\,\varepsilon\,dt \tag{S2}$$

$$I_{net} = |I_+| - |I_-| \tag{S3}$$

$$\varepsilon = (t_+ - t_-)\frac{RT}{F}\ln\frac{\alpha_H}{\alpha_L} \tag{S4}$$

The ion transference number for cations (t_+) and anions (t_-) is defined as,

$$t_+ = \frac{|I_+|}{|I_+| + |I_-|};\ t_- = \frac{|I_-|}{|I_+| + |I_-|} \tag{S5}$$

Then equation S2 can be simplified as,

$$dW = \frac{RT}{F}\ln\frac{a_H}{a_L}\frac{(|I_+| - |I_-|)^2}{|I_+| + |I_-|}\,dt \tag{S6}$$

The physical meaning of equations S1 and S6 is that, the total ion diffusion through the nanopores in an infinitesimal time unit can be characterized as the sum of the absolute value of the diffusion current contributed by cations and anions ($|I_+|+|I_-|$). Whereas, only the net diffusion current ($|I_+|-|I_-|$) generated by the asymmetric ion diffusion across the nanopore can be harvested as electric energy. When the external load equals the internal resistance of the membrane, the energy generation system achieves the maximum output power. In this case, the electric power consumed by the membrane and the external resistance is the same. Thus, the energy conversion efficiency corresponding to the maximum power generation can be calculated as:

$$\eta = \left|\frac{\frac{1}{2}dW}{dG}\right| = \frac{1}{2}\left(\frac{|I_+| - |I_-|}{|I_+| + |I_-|}\right)^2 = \frac{1}{2}(2t_+ - 1)^2 \tag{S7}$$

The calculated energy conversion efficiency is listed in Table S1.

7. Energy conversion under different electrolyte conditions.

The osmotic energy conversion in V-GO can be adjusted by the pH value of electrolyte solution. Alkaline solution can effectively increase power generation because the presence of OH^- promotes the ionization of carboxyl group in the surface of GO sheet and accordingly enhances the ion selectivity of the V-GO (Figure S15**,** Supporting Information), which consistent with previous literature reports.[2] Similarly, the suppression of ionization of carboxyl group in acidic solutions decreases the surface charge density of GO sheet. Thus, the membrane potential is the lowest in the solution with the pH value of 3. Besides the pH value, the rise of concentration difference also enhances membrane potential due to the intense diffusion under high concentration gradient.

8. Membrane thickness dependent energy conversion.

The tailor-made V-GO with different thicknesses were fixed between a pair of electrolyte solution cells. By mixing artificial seawater and river water, a source meter (Keithley 6487) was used to measure the output current. The output power (P_R) can be obtained by $P_R = I^2 \times R_L$. The output power density decreases linearly with the increase of membranes thickness in Figure S16. It shows that the energy conversion of V-GO has classical Ohm-like dependence. Specifically, the increment of membrane thickness linearly reduces the diffusion current; and it does not affect the membrane potential, because the change of membrane thickness does not affect the ion selectivity.

9. Membrane area dependent energy conversion.

By enlarging the effective membrane area, the total output power of V-GO can be easily promoted. What is important, the expansion of membrane area does not impair the power density. As shown in Figure S17, the prolonged width (W) raises the diffusion current linearly; and it does not affect the membrane potential, since the change in membrane area will not affect the ion selectivity. Thus, the output power can be linearly improved by enlarging the membrane area. It is beneficial for the scaling up of V-GO as the osmotic energy conversion systems.

10. Ion selectivity and permeation.

We performed more ionic conduction experiment with V-GO samples. The measured ionic current suggests Mg^{2+} ions can transport through the V-GO as well as Na^+ ions (Figure S18).

The energy conversions tested in more salts, including LiCl, NaCl and KCl, indicate that higher mobility of cations can effectively enhance the diffusion current and membrane potential (Figure S19).

The ion selectivity of H-GO is tested by the similar method with V-GO. When the ionic current is measured under asymmetric concentration condition (1 M |1 μM NaCl), the major ionic carriers across the membrane come from the left reservoir. Therefore, the positive and negative ionic currents are dominated by Na^+ and Cl^-, respectively. The ion selectivity can be evaluated by the cation transference number (t_+) ($t_+ = |I_+|/(|I_+|+|I_-|)$). I_+ and I_- are the ion flux contributed by cations and anions respectively. For example, in V-GO, the tested Na^+ current is 10.9 times higher than the Cl^- current. Thus, the cation transference number can be calculated through t_+ = 10.9/(10.9+1)= 0.916. This method has been adopted to scale the charge selectivity of nanoporous membranes.[7] Similarly, the cation transference number of H-GO was calculated to be 0.909 (Figure S20).

11. MD simulation.

To construct the MD simulation model of GO, 7.5 nm × 4 nm graphene sheets were prepared with one 1.5 nm-wide gap on the left or right edge (Figure S21, Supporting Information). They were added with randomly distributed epoxy and hydroxyl functional groups on both sides by following the Lerf−Klinowski model (large fractions of hydroxyl and epoxy were bonded to carbon atoms next to each other.[8,9] All edge sites were functionalized with evenly spaced carboxylic acid groups and hydrogen atoms at the liner charge density of 2 e nm^{-1}.[10] One exception is that no charge was added on the outside edges of H-GO to prevent the ion from leaking through when applying the periodic boundary condition. The final element ratio C:H:O=52 : 16 : 32 in V (H)-GO and the surface charge density is 0.53 e nm^{-2} in H-GO, which is consistent with the experimental values. Four layers of functionalized GO were stacked to form a GO with the interlayer spacing of 1 nm. The simulation box was 4×4×20 nm^3 (7.6×4×9 nm^3) in V (H)-GO. The concentration of NaCl solutions on both sides of the GO was the same as 1 M and the whole system was charge neutralized.

In the MD simulation, a 0.7 V nm^{-1} voltage bias was applied along the migration direction of the ions through GO to accelerate simulations. Since the simulation model of GO is only several nanometers in size, which is much smaller than the experimental system in hundreds of micrometers, a periodic boundary condition is applied along all directions. Loading time was the time elapsed between two ions (which can go inside the GO at least 0.8 nm) sequentially entering the GO. The passing velocity was calculated by dividing the channel

length with the average transit time.

As shown in Figure S22a, the ions passing through H-GO have to take zigzag trajectories to go through the gaps between adjacent layers. In sharp contrast, most of the ions can pass straight through a single channel inside V-GO (Figure S22b). Therefore, the ion average velocity along the permeation direction across H-GO is remarkably slower than that in V-GO. Moreover, the tortuous geometric structures in H-GO produce a strong barrier to impede the access of Na^+. It results in the evident ion enrichment at the entrance of H-GO (Figure S23).

Supporting Figures and Tables

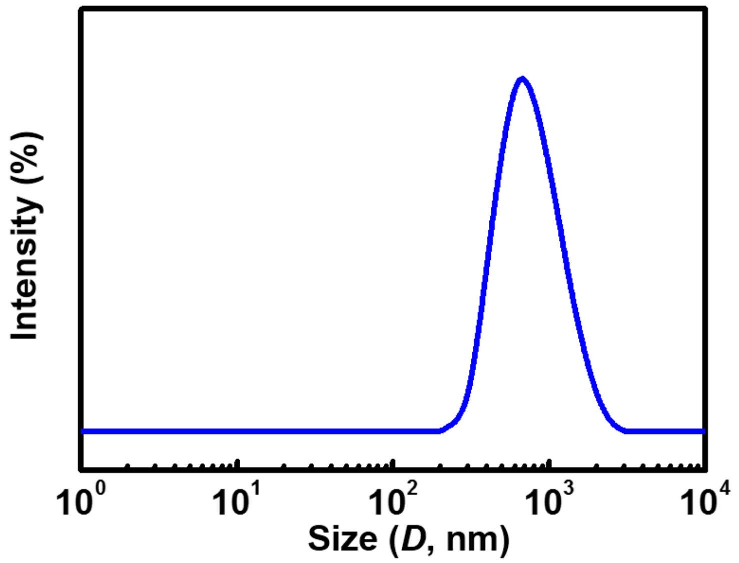

Figure S1. Size distribution of GO colloids was observed by a Malvern Zetasizer NanoZS90. The sizes of GO sheets ranges from 400 nm to 1000 nm. The concentration of GO dispersion is 0.1 mg mL^{-1}.

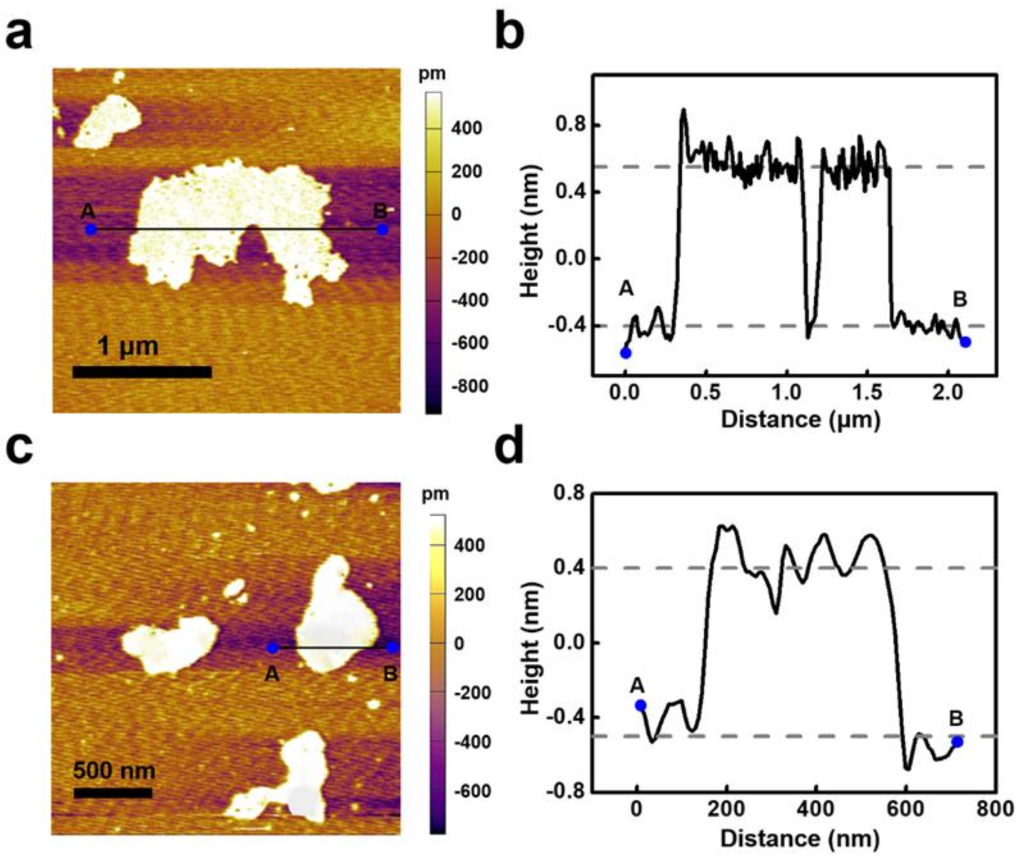

Figure S2. Representative AFM images show that the height of individual GO sheets is 0.9±0.1 nm.

.

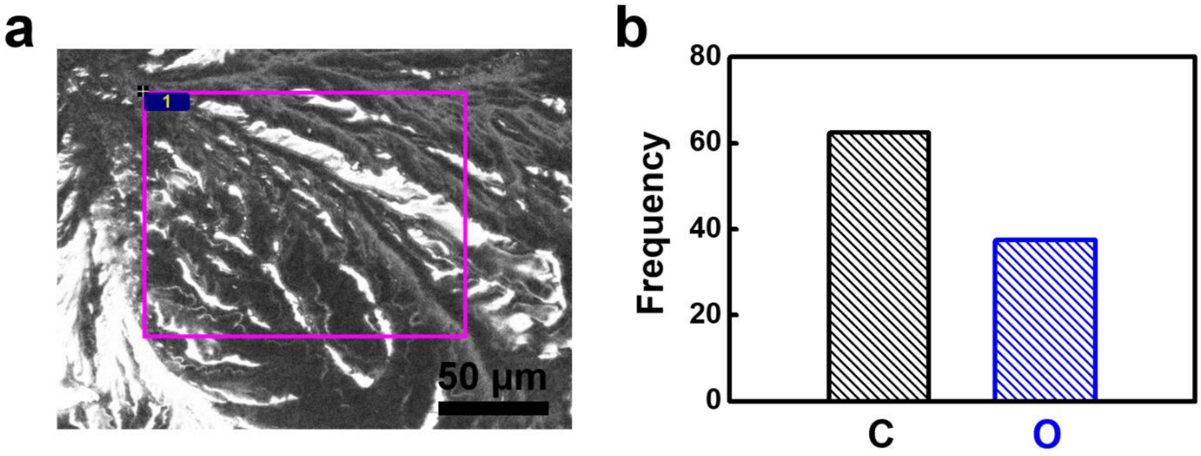

Figure S3. a) SEM observations on the surface of GO. b) The corresponding EDS analysis in the zone 1 from a). The graphite was partially oxidized to oxygen-containing groups.

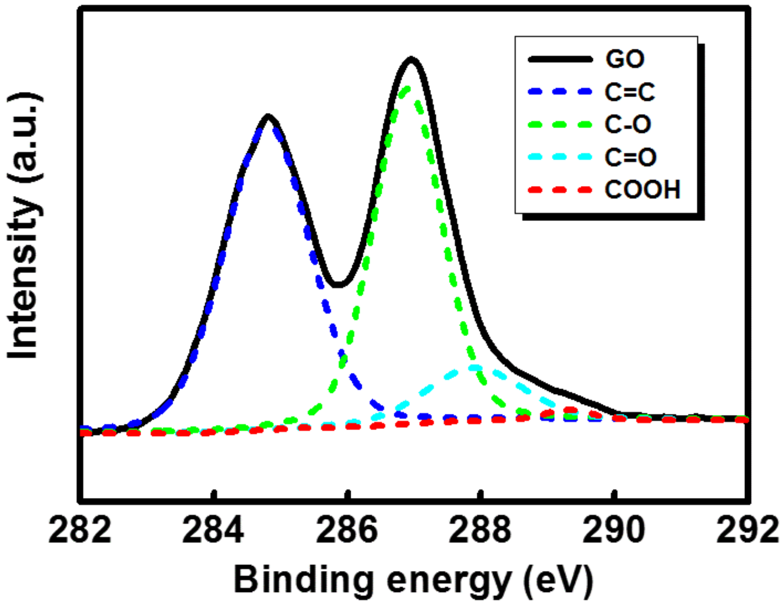

Figure S4. XPS analysis of GO. The composite peak of GO (solid line) was decomposed into four individual peaks (dashed lines) including C=C (284.8 eV), C-OH (286.9 eV), C=O (287.9 eV), and COOH (289.3 eV). The percentage of carboxylic acid group is 1.2% of the total carbon content.

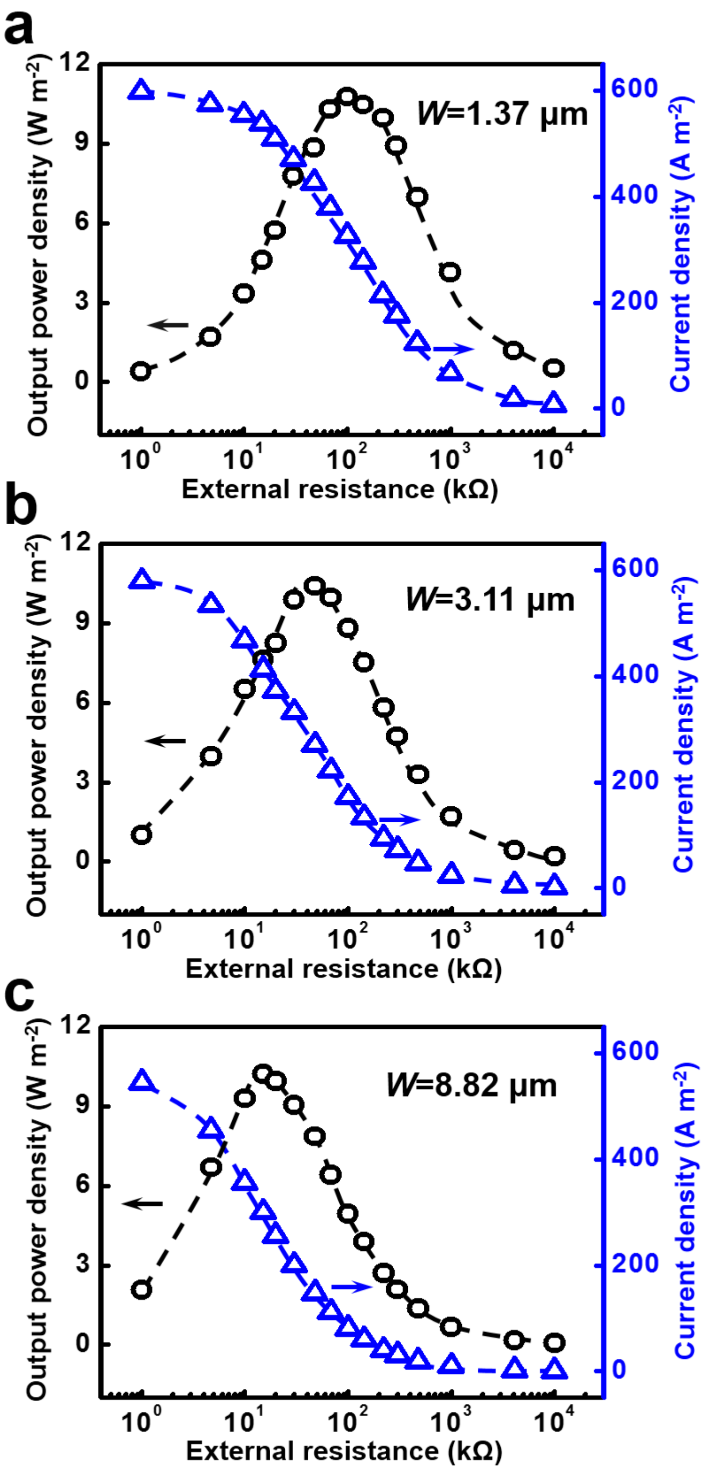

Figure S5. The output power densities and current densities measured in the external load when the areas of V-GO are a) 1.37 μm × 0.98 mm, b) 3.11 μm × 0.96 mm, and c) 8.82 μm × 1.02 mm, respectively. Diluted (LC) and concentrated (HC) NaCl solution remains at 10 mM and 500 mM, respectively. These results correspond to Figure 2c.

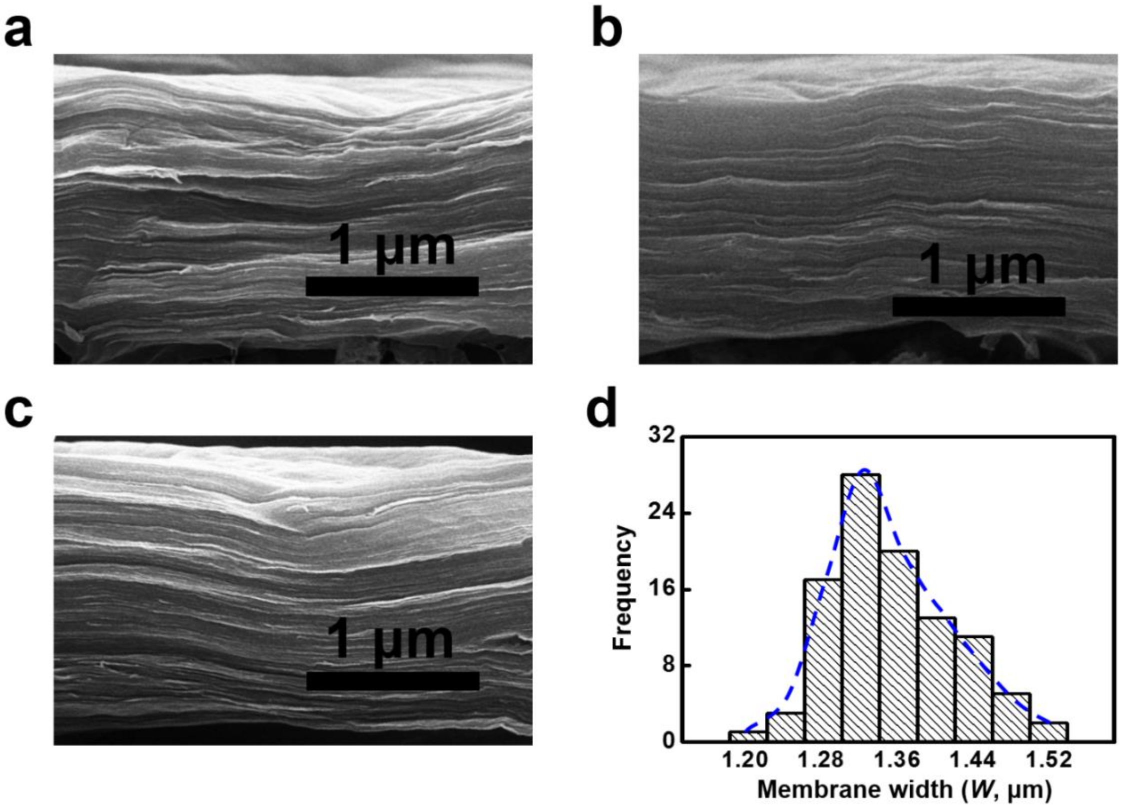

Figure S6. SEM observations to measure average width of the cross section of V-GO. a,b, c) The typical SEM images of the cross section in V-GO with the width (*W*) of 1.37 µm. d) Statistical results over 100 samples show that the width distribution of V-GO is 1.37 ± 0.17 µm.

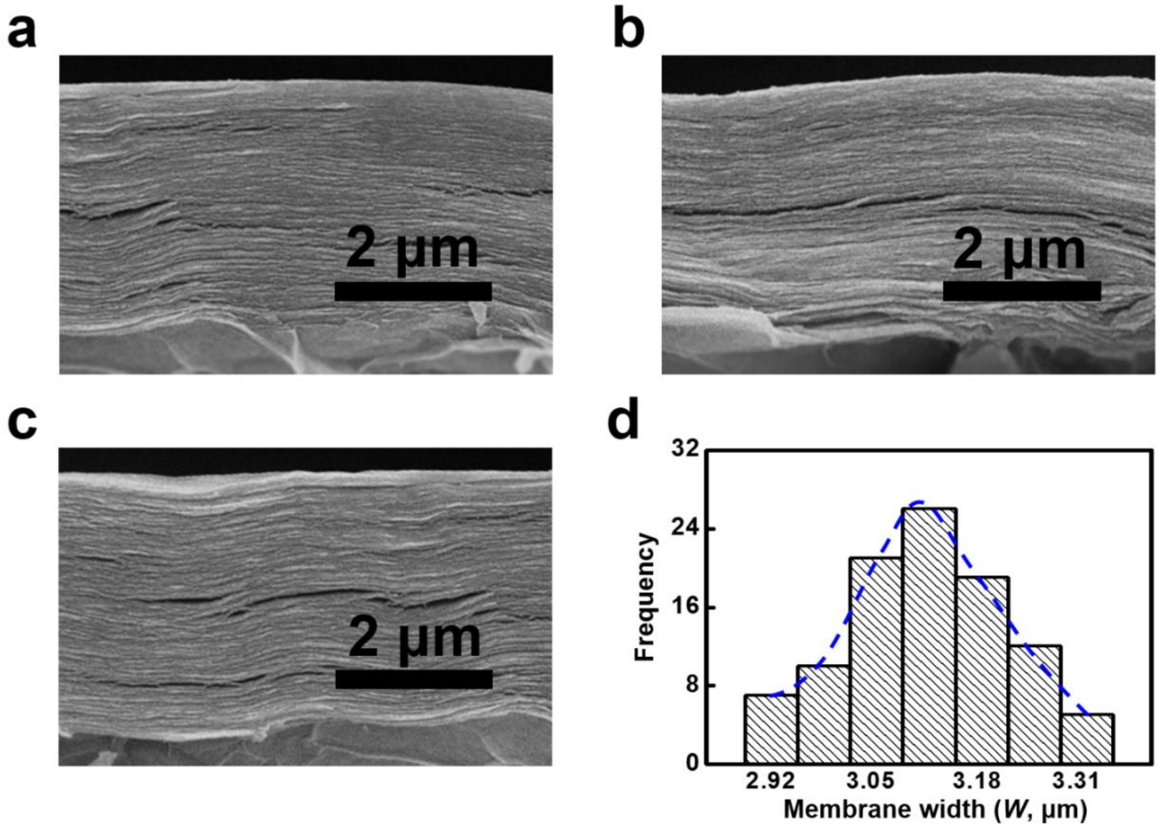

Figure S7. SEM observations to measure average width of the cross section of V-GO. a, b, c) The typical SEM images of the cross section in V-GO with the width (*W*) of 3.11 μm. d) Statistical results over 100 samples show that the the width distribution of V-GO is 3.11 ± 0.19 μm.

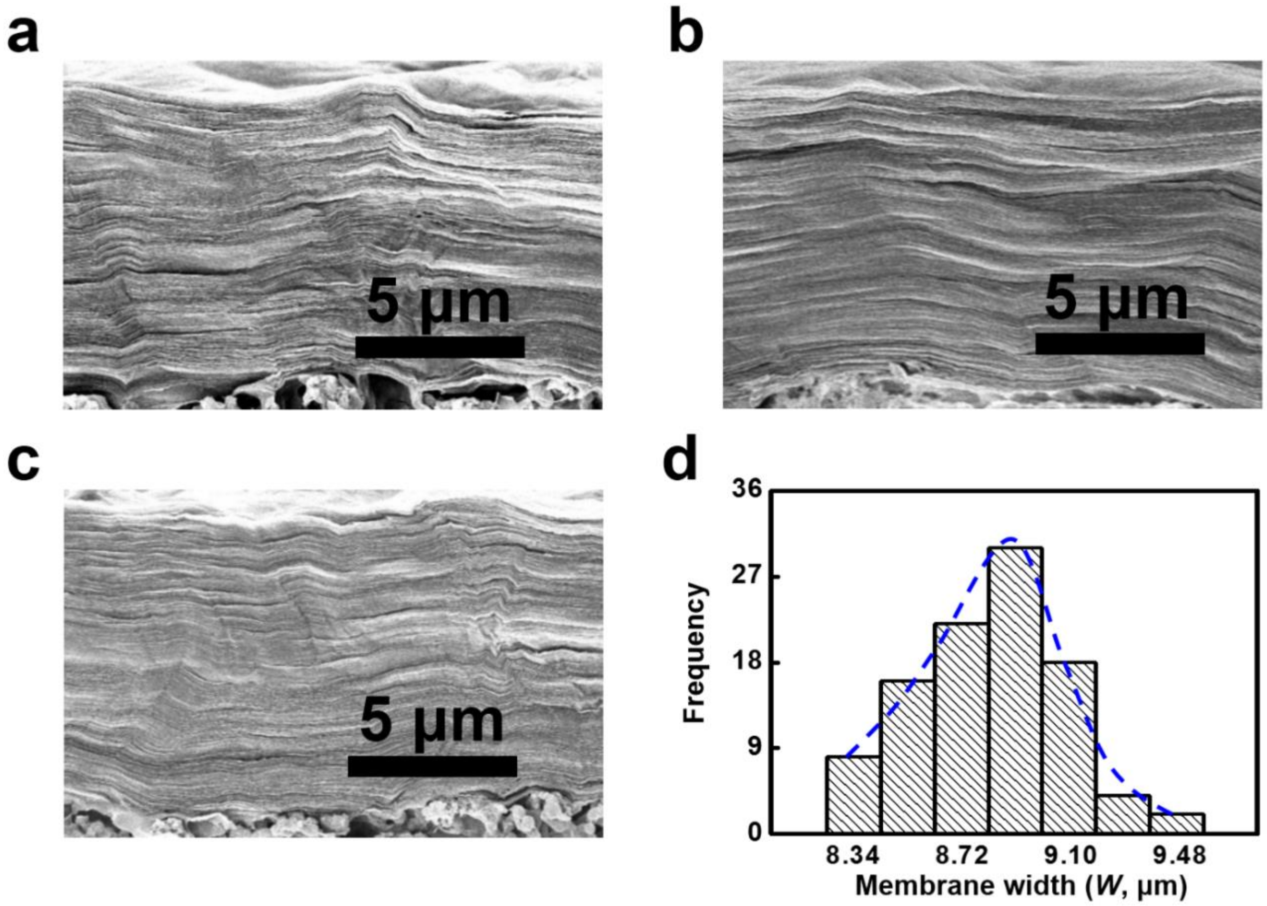

Figure S8. SEM observations to measure average width of the cross section of V-GO. a, b, c) The typical SEM images of the cross section in V-GO with the width (*W*) of 8.82 μm. d) Statistical results over 100 samples show that the the width distribution of V-GO is 8.82 ± 0.48 μm.

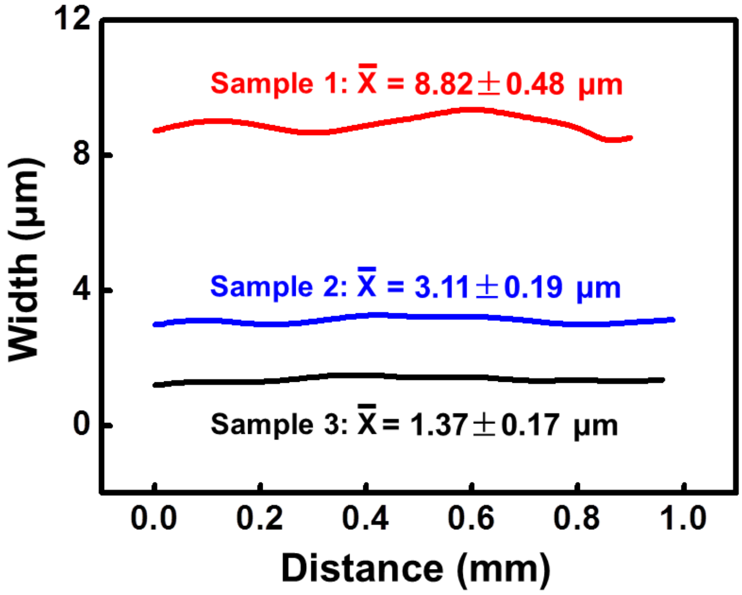

Figure S9. The width uniformity of different samples.

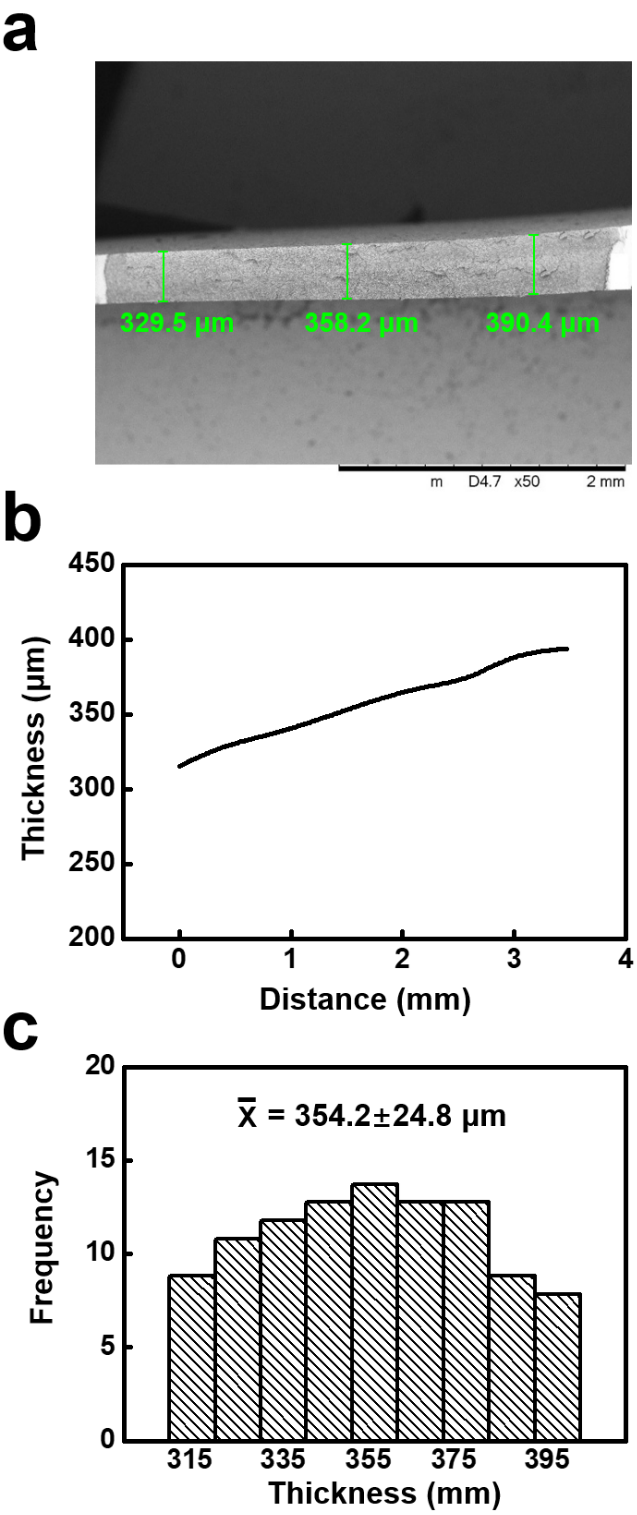

Figure S10. The thicknesses of the tested V-GO samples were measured by SEM. a) Typical SEM image to test the membrane thickness. b) We measure 100 data throughout all ~1 mm length in the sample. The data was ploted together to obtain the thickness uniformity of the sample. c) The average value of membrane thickness was calculated to be 354.2±24.8 μm through statistical method.

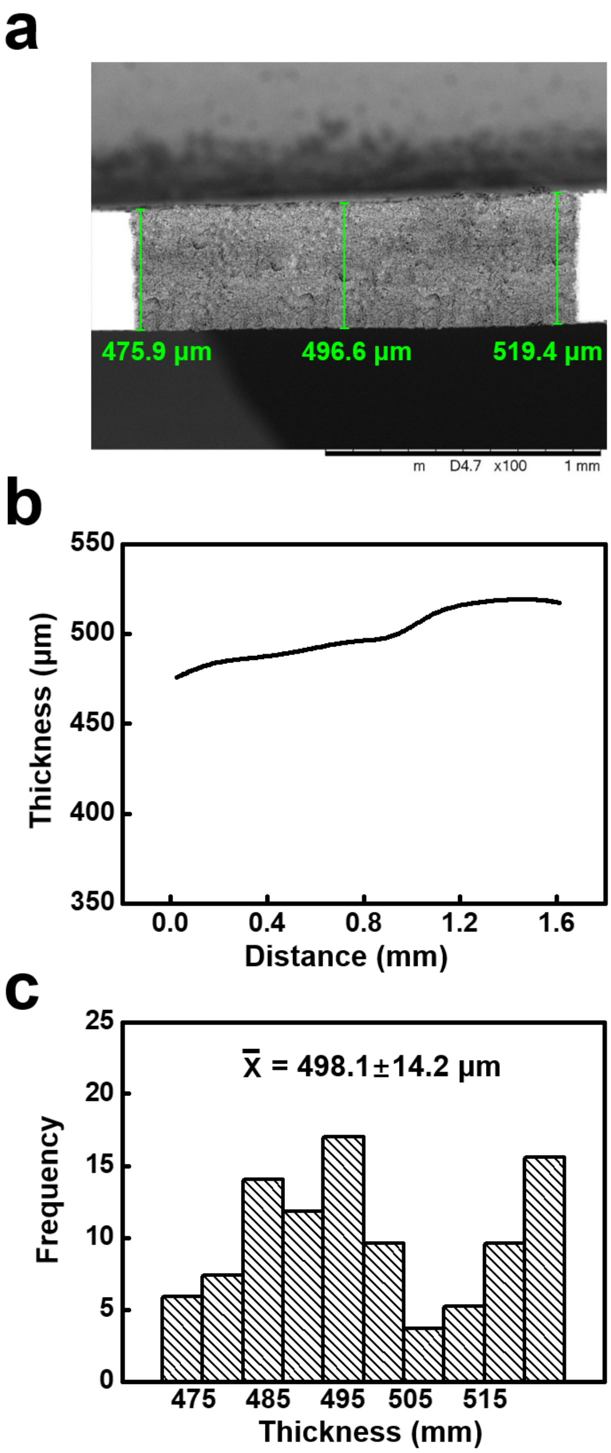

Figure S11. The thicknesses of the tested V-GO samples were measured by SEM. a) Typical SEM image to test the membrane thickness. b) We measure 100 data throughout all ~1 mm length in the sample. The data was ploted together to obtain the thickness uniformity of the sample. c) The average value of membrane thickness was calculated to be 498.1±14.2 μm through statistical method.

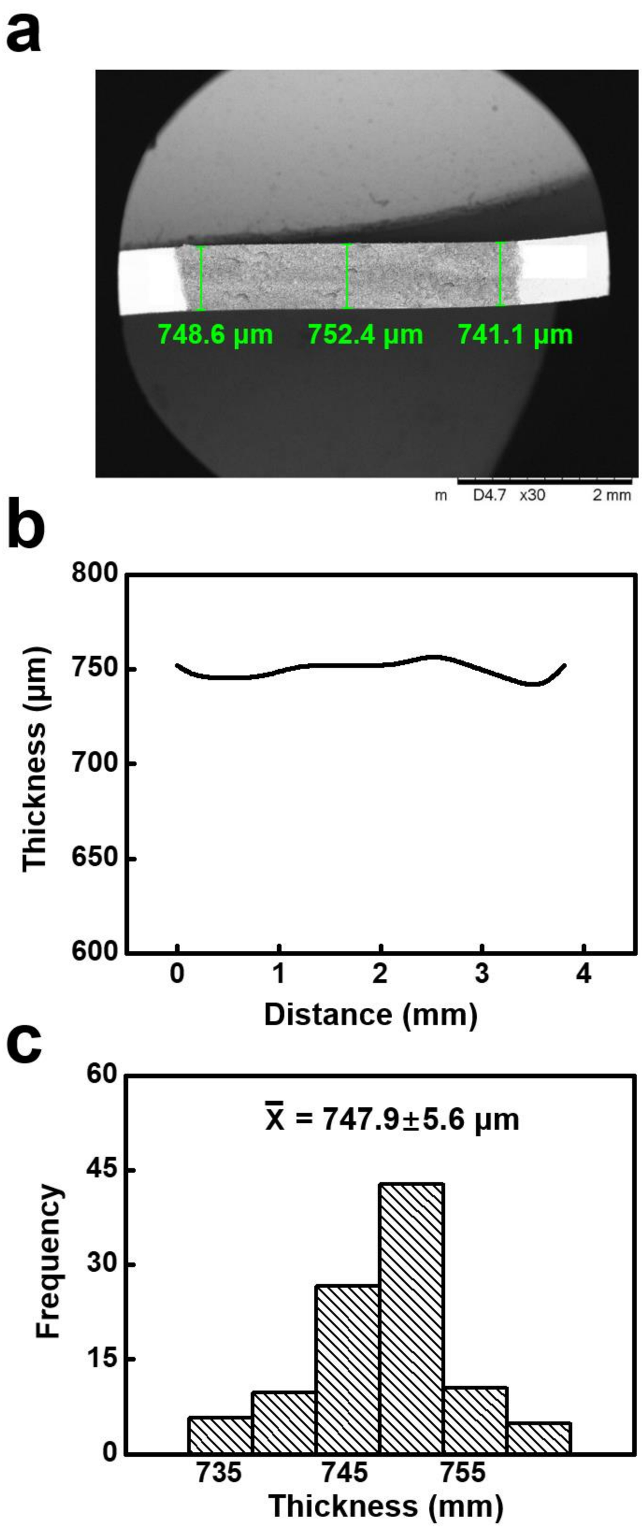

Figure S12. The thicknesses of the tested V-GO samples were measured by SEM. a) Typical SEM image to test the membrane thickness. b) We measure 100 data throughout all ~1 mm length in the sample. The data was plot together to obtain the thickness uniformity of the sample. c) The average value of membrane thickness was calculated to be 747.9±5.6 μm through statistical method.

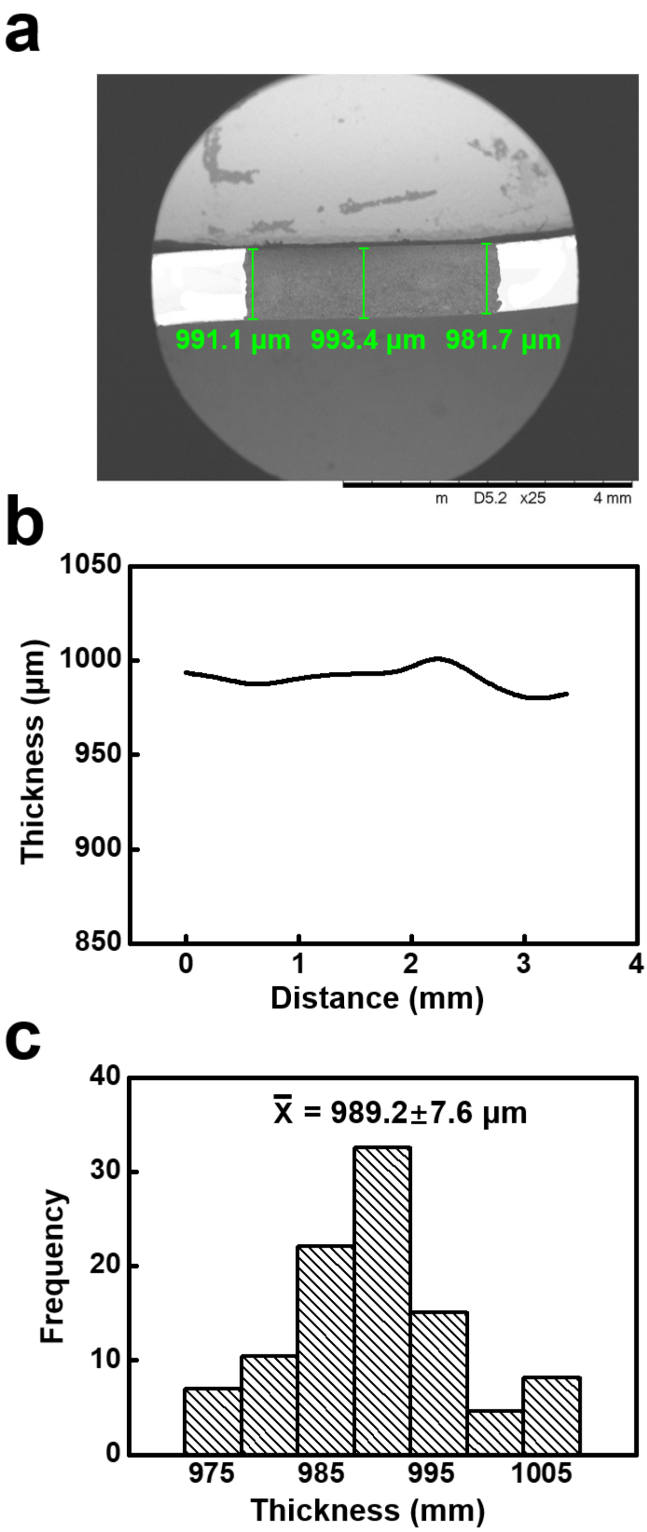

Figure S13. The thicknesses of the tested V-GO samples were measured by SEM. a) Typical SEM image to test the membrane thickness. b) We measure 100 data throughout all ~1 mm length in the sample. The data was plot together to obtain the thickness uniformity of the sample. c) The average value of membrane thickness was calculated to be 989.2±7.6 µm through statistical method.

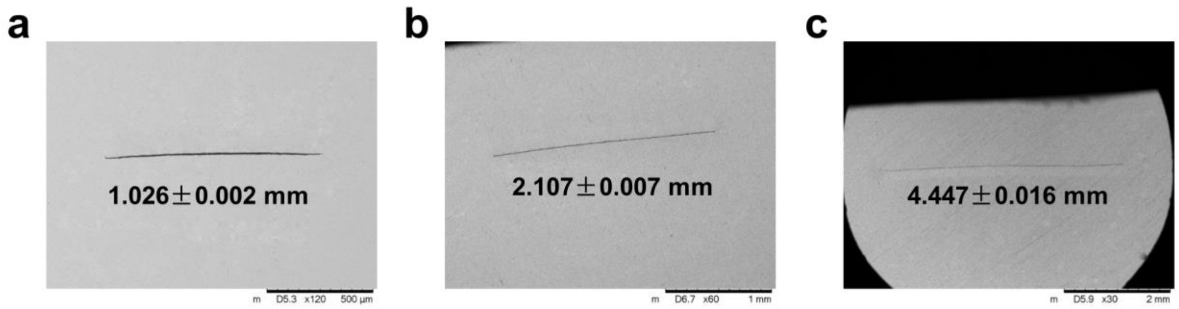

Figure S14. SEM observations to measure the length (*L*) of the cross section of V-GO. a, b, c) The typical SEM images of the cross section in V-GO samples. The length of V-GO is 1.026±0.002 mm, 2.107±0.007 mm, and 4.447±0.016 mm, respectively.

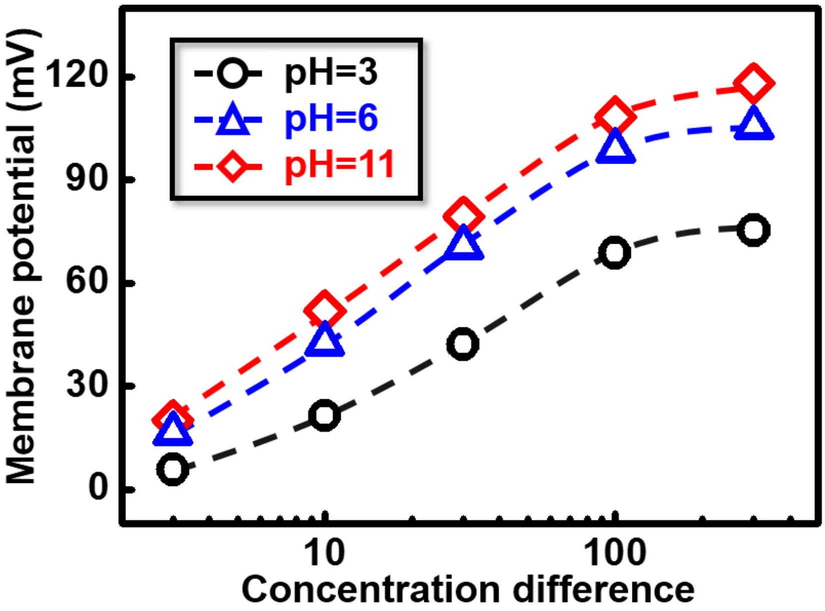

Figure S15. The membrane potential of V-GO increases with the pH value and ion concentration difference.

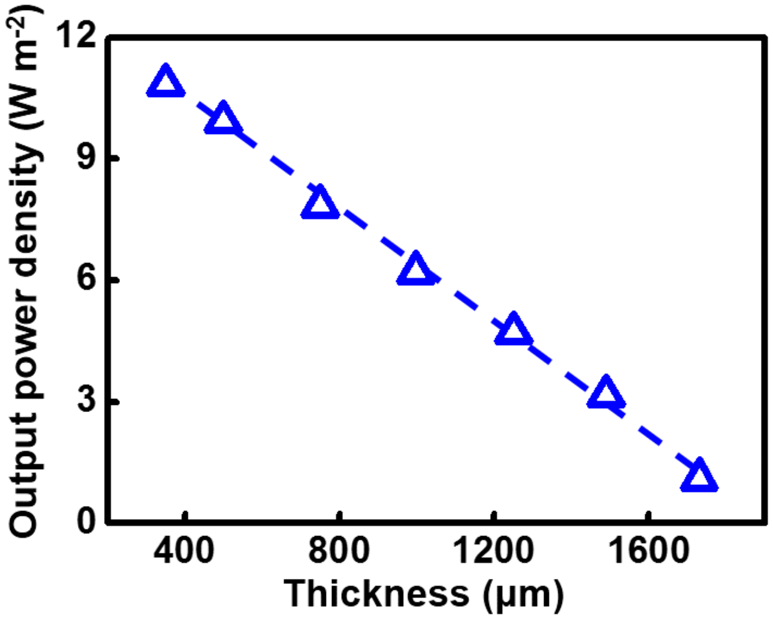

Figure S16. Under 50-fold concentration difference of NaCl solution, the output power density linearly decreases with the increment of the thickness of V-GO samples.

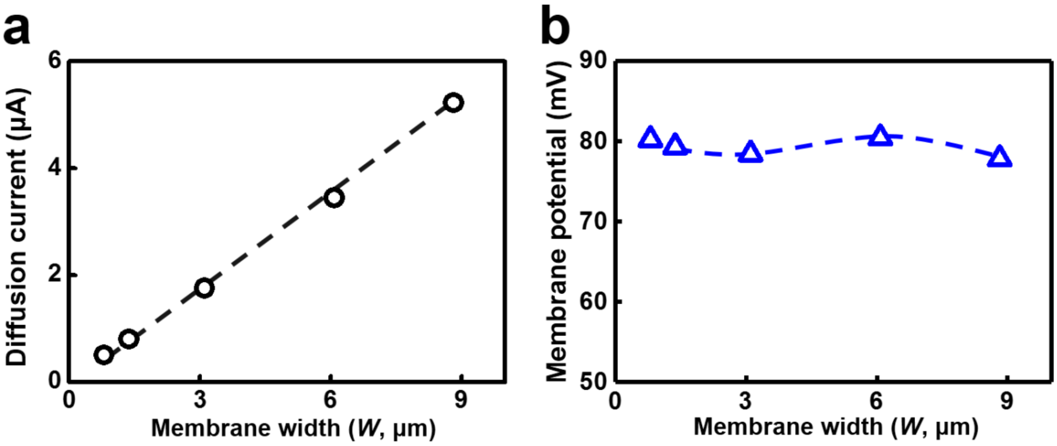

Figure S17. a) The prolonged width (*W*) raises the diffusion current linearly. b) The membrane potential basically maintains at 80 mV while the the widths of V-GO samples ranges from 0.5 μm to 9 μm. The applied solution is 50-fold NaCl solution.

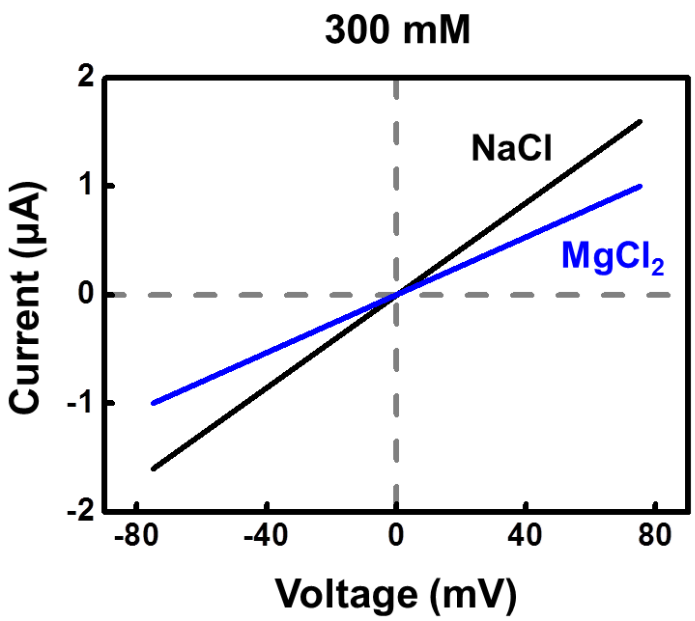

Figure S18. The IV curve tested in $MgCl_2$ solution indicates the Mg^{2+} can also go through the V-GO.

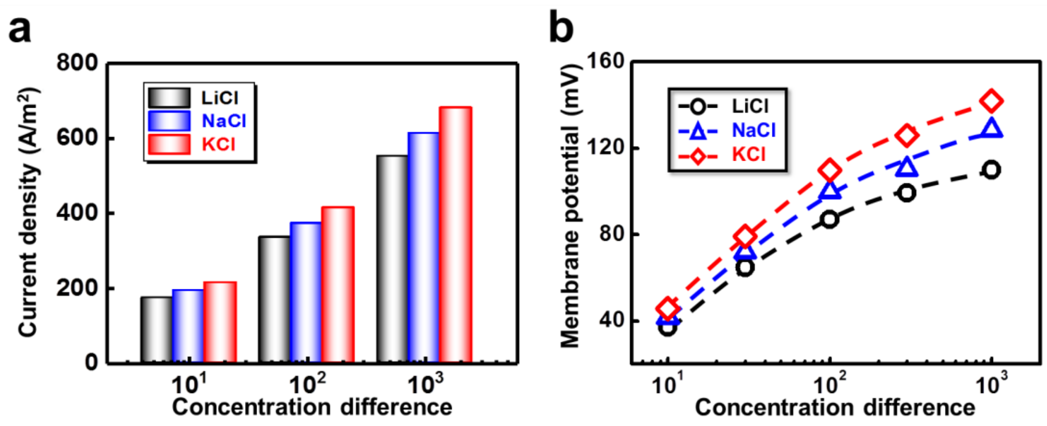

Figure S19. The energy conversions tested in more salts, including LiCl, NaCl and KCl, indicate that higher cation mobility can effectively enhance the diffusion current and membrane potential.

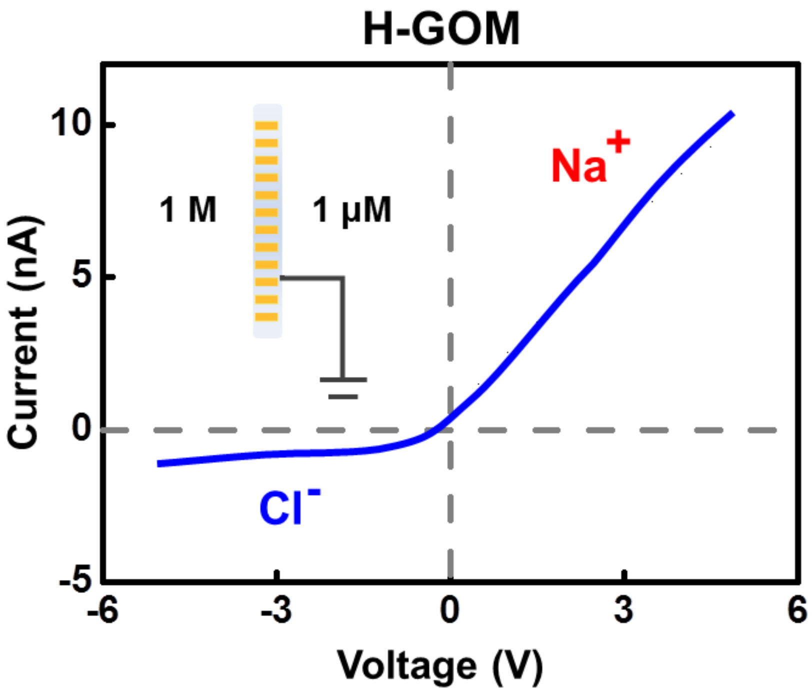

Figure S20. Ion selectivity of the H-GO is tested through the ionic current under asymmetric concentration. The condition is 1 M |1 μM NaCl. The cation transference number (t_+) is calculated to be 0.909.

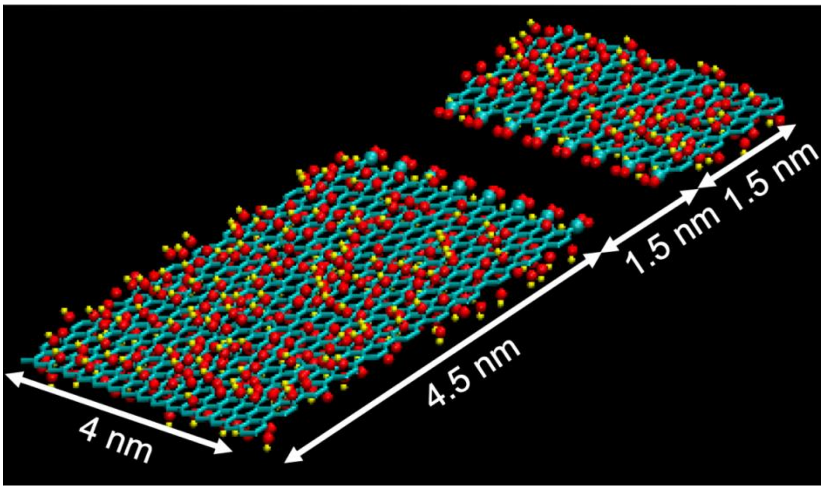

Figure S21. The Simulation model of a single slit of GO flakes. Each GO flake (7.5 × 4 nm^2) is generated by adding functional groups in a graphene sheet. The graphitic backbone of GO is shown in cyan lines; and carbon, oxygen and hydrogen atoms are shown in cyan, red and yellow spheres, respectively.

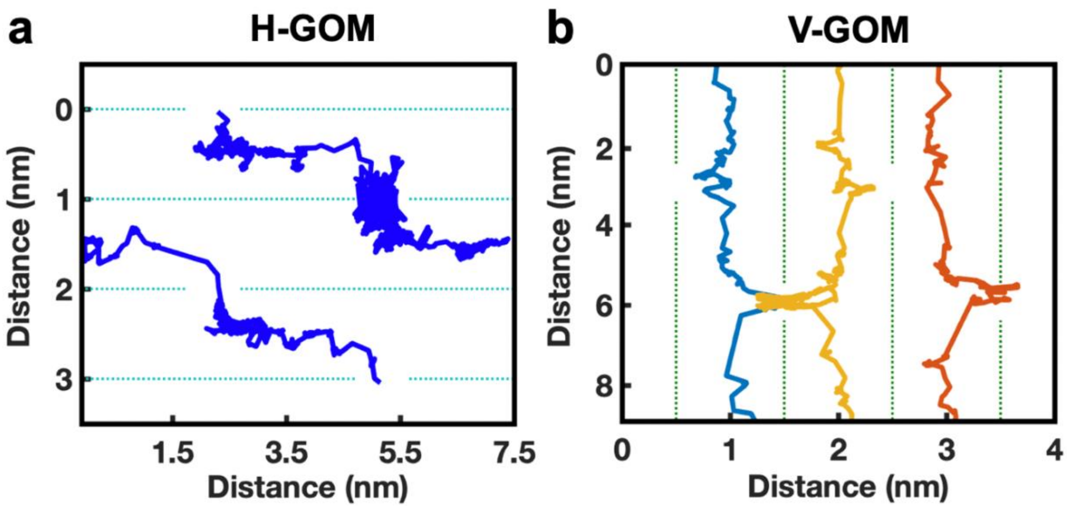

Figure S22. Representative transport trajectories of Na^+ ions in a) H-GO and b) V-GO. Na^+ take zigzag trajectories (solid line) to go through the gaps between adjacent GO layers (dotted lines) in H-GO, but pass through a single channel between adjacent GO layers (dotted lines) inside V-GO.

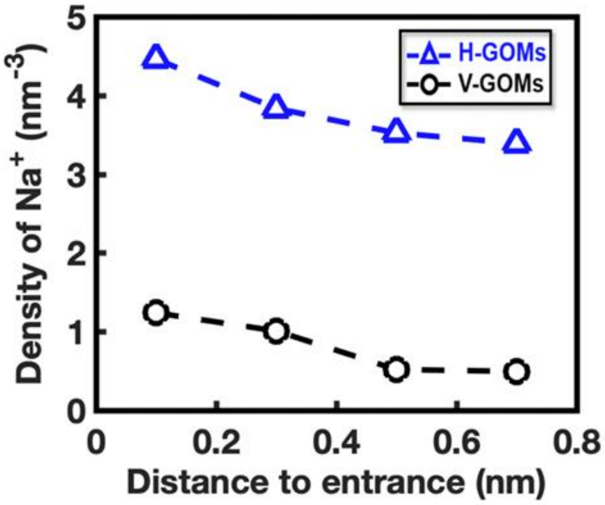

Figure S23. Comparison of the average density of Na^+ ions at the entrance region as a function of the distance from the entrance of H-GO and V-GO.

Table S1. Energy conversions in different membranes under artificial seawater and river water conditions. *U, I* and P_{max} stand for membrane potential, diffusion current and maximum output power density, respectively.

Membrane type	Experimental condition	U (mV)	I (A m^{-2})	P_{max} (W m^{-2})	Thickness (μm)	Efficiency (%)	Ref.
MKNCM	0.5 M | 0.01 M NaCl	-	-	3.92	2	35	[11]
UFSCNM	0.1 M | 0.0001 M KCl	76	12	0.21	0.25	9.2	[12]
PSS/MOF	0.5 M | 0.01 M NaCl	70	170	2.87	1.6	24.4	[13]
H-GO	0.5 M | 0.01 M NaCl	160	20	0.77	10	36.6	[2]
PET-BCP	0.5 M | 0.01 M NaCl	75.9	20	0.35	13.5	28.5	[14]
FKS	0.5 M | 0.01 M NaCl	100.2	12.5	0.26	20	49.7	[15]
PPy	0.5 M | 0.01 M NaCl	73.4	4.9	0.087	20	26.7	[16]
PCTE	1 M | 0.001 M KCl	55	4.74	0.058	20	4.8	[17]
IDM	0.5 M | 0.01 M NaCl	73.2	215.7	3.46	64	26.5	[15]
CMI	0.5 M | 0.01 M NaCl	99.1	14.2	0.4	320	48.6	[15]
This work	0.5 M | 0.01 M NaCl	81.3	570	10.6	358	34.6	-

Table S2. Osmotic energy conversion of V-GO outperformances all reported materials under various conditions.

Membrane type	Experimental condition	P_{max} (W m^{-2})	Thickness (μm)	Ref.	V−GO (W m^{-2})
UFSCNM	0.1 M ∣ 0.0001 M KCl pH=6	0.21	0.25	[12]	14.3
GRED	1 M ∣ 0.001 M NaCl pH=6	8	~12	[18]	19.5
SNF-AAO	0.5 M ∣ 0.01 M NaCl pH=11	2.86	90-110	[19]	13.6
$Ti_3C_2T_x$ MXene	1 M ∣ 0.001 M KCl pH=11	20.85	2.7	[20]	33.6
Silica nanochannels	1 M ∣ 0.001 M KCl pH=6	7.7	140	[21]	23.4
PES-Py/PAEK-HS	5 M ∣ 0.01 M NaCl pH=6	5.1	11	[22]	32.1

References

[1] H. Huang, Z. Song, N. Wei, L. Shi, Y. Mao, Y. Ying, L. Sun, Z. Xu, X. Peng, *Nat. Commun.* **2013**, *4*, 2979.
[2] J. Ji, Q. Kang, Y. Zhou, Y. Feng, X. Chen, J. Yuan, W. Guo, Y. Wei, L. Jiang, *Adv. Funct. Mater.* **2017**, *27*, 1603623.
[3] D. A. Dikin, S. Stankovich, E. J. Zimney, R. D. Piner, G. H. Dommett, G. Evmenenko, S. T. Nguyen, R. S. Ruoff, *Nature* **2007**, *448*, 457.
[4] L. Chen, B. Peng, J. Wang, D. Li, Z. Qian, Y. Yang, W. Jin, L. Chen, G. Shi, B. Zhang, Y. Wang, F. Bian, D. Li, G. Liu, J. Zeng, M. Wu, J. Li, H. Fang, L. Chen, L. Zhang, J. Shen, G. Xu, G. Zhou, *Nature* **2017**, *550*, 1.
[5] N. A. Kotov, I. Dekany, J. H. Fendler, *Adv. Mater.* **1996**, *8*, 637.
[6] W. Guo, L. Cao, J. Xia, F.-Q. Nie, W. Ma, J. Xue, Y. Song, D. Zhu, Y. Wang, L. Jiang, *Adv. Funct. Mater.* **2010**, *20*, 1339.
[7] J. Gao, W. Guo, D. Feng, H. Wang, D. Zhao, L. Jiang, J. Am. Chem. Soc. **2014**, *136*, 12265.
[8] A. Lerf, H. He, M. Forster, J. Klinowski, *J. Phys. Chem. B* **1998**, *102*, 4477.
[9] H. Dai, Z. Xu, X. Yang, *J. Phys. Chem. C* **2016**, *120*, 22585.
[10] C. D. Williams, P. Carbone, F. R. Siperstein, *Nanoscale* **2018**, *10*, 1946.
[11] Z. Zhang, S. Yang, P. Zhang, J. Zhang, G. Chen, X. Feng, *Nat. Commun.* **2019**, *10*, 2920.
[12] K. Xiao, P. Giusto, L. Wen, L. Jiang, M. Antonietti, *Angew. Chem. Int. Ed.* **2018**, *57*, 10123.
[13] R. Li, J. Jiang, Q. Liu, Z. Xie, J. Zhai, *Nano Energy.* **2018**, *53*, 643.
[14] Z. Zhang, X. Y. Kong, K. Xiao, Q. Liu, G. Xie, P. Li, J. Ma, Y. Tian, L. Wen, L. Jiang, *J. Am. Chem. Soc.* **2015**, *137*, 14765.
[15] J. Gao, W. Guo, D. Feng, H. Wang, D. Zhao, L. Jiang, *J. Am. Chem. Soc.* **2014**, *136*, 12265.
[16] C. Yu, X. Zhu, C. Wang, Y. Zhou, X. Jia, L. Jiang, X. Liu, G. G. Wallace, *Nano Energy.* **2018**, *53*, 475.
[17] K. Kwon, S. J. Lee, L. Li, C. Han, D. Kim, *Int. J. Energ Res.* **2014**, *38*, 530.
[18] Y. Fu, X. Guo, Y. Wang, X. Wang, J. Xue, *Nano Energy*. **2019**, *57*, 783.
[19] W. Xin, Z. Zhang, X. Huang, Y. Hu, T. Zhou, C. Zhu, X.-Y. Kong, L. Jiang, L. Wen, *Nat. Commun.* **2019**, *10*, 3876.
[20] S. Hong, F. Ming, Y. Shi, R. Li, I. S. Kim, C. Y. Tang, H. N. Alshareef, P. Wang, *ACS Nano.* **2019**, *13*, 8917.
[21] D.-K. Kim, C. Duan, Y.-F. Chen, A. Majumdar, *Microfluid. Nanofluid.* **2010**, *9*, 1215.
[22] X. Zhu, J. Hao, B. Bao, Y. Zhou, H. Zhang, J. Pang, Z. Jiang, L. Jiang, *Sci. Adv.* **2018**, *4*, eaau1665.
